# Reducing photosensitizer aggregation *via* cyclodextrin–adamantane porphyrin host–guest interactions for enhanced photodynamic therapy

**DOI:** 10.1039/d6ra05237a

**Published:** 2026-10-27

**Authors:** Chengcheng Liu, Lijun Yue, Chuning Jia, Yelin Wu, Chaochao Wang

**Affiliations:** a School of Medicine, Anhui University of Science and Technology Huainan 232001 P. R. China; b Institute of Hepatobiliary and Pancreatic Surgery, Department of Hepatobiliary and Pancreatic Surgery, Shanghai East Hospital, School of Medicine, Tongji University Shanghai 200120 P. R. China sk_wuyelin@tongji.edu.cn wcchao@tongji.edu.cn

## Abstract

The production yield of singlet oxygen (^1^O_2_) directly determines the therapeutic efficacy of photodynamic therapy (PDT). However, photosensitizer aggregation seriously suppresses ^1^O_2_ production. Herein, we developed a host–guest complexation-based isolation strategy that prevents photosensitizers' aggregation, enhances ^1^O_2_ generation and ultimately boosts the photodynamic killing performance. Specifically, we synthesized ultra-small Cu_2−*x*_Se-CD nanoparticles as a supramolecular loading platform and loaded them with adamantane-functionalized 5-(4-hydroxyphenyl)-10,15,20-triphenylporphyrin (Ada-TPP) to obtain a supramolecular nano-photosensitizer Cu_2−*x*_Se-CD-TPP. In this construct each photosensitizer molecule is individually confined within the cavity of a cyclodextrin unit anchored on the surface of Cu_2−*x*_Se nanoparticles, endowing the material with excellent dispersibility and effectively suppressing the photosensitizer's aggregation. This rational structural design results in an 84.8% increase in ^1^O_2_ production yield. Both *in vitro* and *in vivo* experiments confirm that Cu_2−*x*_Se-CD-TPP achieves efficient tumor therapy under light irradiation. Collectively, this study not only provides a robust strategy to mitigate photosensitizers' aggregation, but also develops a promising nanomaterial for precise and high-efficacy photodynamic therapy.

## Abstract 2

A host–guest complexation-based isolation strategy was proposed to suppress the aggregation of photosensitizers. This structural isolation improves singlet oxygen production, thereby boosts the photodynamic therapeutic efficacy against tumor cells.

## Graphical abstract

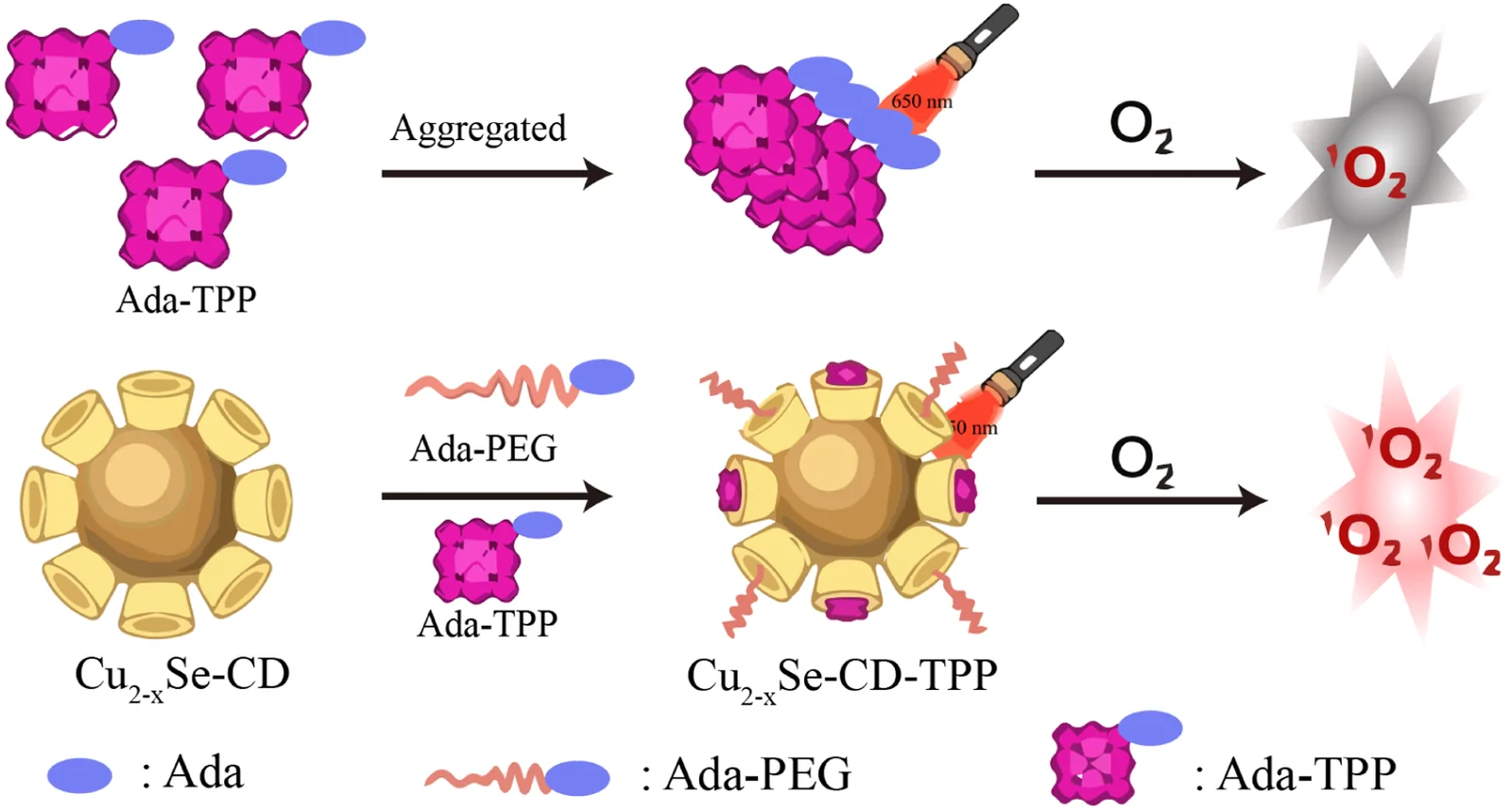

## 1. Introduction

Photodynamic therapy (PDT) has been widely regarded as an attractive and effective cancer treatment modality in recent decades. It mainly kills tumor cells by utilizing singlet oxygen generated by a photosensitizer under light irradiation.^1,2^ Thus, the yield of singlet oxygen directly determines the therapeutic efficacy of PDT. Porphyrin-based photosensitizers are among the most extensively studied and clinically employed photosensitizers for PDT.^3,4^ However, porphyrin photosensitizers possess strong hydrophobicity and a large π-conjugated system, which lead to their aggregation in the aqueous physiological environment.^5,6^ This aggregation causes photosensitizers to dissipate energy as heat or superoxide anions upon light irradiation, while the quantum yields of ^1^O_2_ are significantly reduced.^7,8^ Worse, the generated heat is insufficient to raise the temperature of tumor tissues to a lethal level, and the superoxide anions produced are rapidly cleared by high levels of SOD in the tumor microenvironment.^9,10^ Therefore, the aggregation of photosensitizers severely impairs singlet oxygen-dependent PDT.

To address the aggregation of porphyrin-based photosensitizers, a variety of strategies have been developed, which generally fall into three categories: improving intrinsic water solubility *via* chemical modification (*e.g.*, grafting hydrophilic components such as glutathione or PEG onto the photosensitizer backbone),^11–13^ designing responsive photosensitizers for the tumor microenvironment, and introducing steric barriers to suppress intermolecular aggregation (*e.g.*, constructing POSS–porphyrin alternating copolymers).^14,15^ Although these strategies have achieved progress in inhibiting photosensitizer aggregation, they still face notable limitations when applied to many common porphyrin photosensitizers. Major limitations include insufficient steric isolation of photosensitizer molecules, incomplete stimuli-responsiveness, and severe premature leakage during systemic delivery.^16–18^ Accordingly, developing general and effective novel strategies to prevent porphyrin photosensitizer aggregation is critically important for advancing the efficacy of PDT.

Cyclodextrins (CDs) are a class of cyclic oligosaccharides, typically consisting of 6–12 d-glucopyranose units. Among them, CDs containing 6, 7, or 8 sugar units are defined as α-, β-, and γ-cyclodextrins, respectively.^19,20^ These CDs possess a unique structure: a hydrophobic inner cavity and a hydrophilic outer surface, rendering them water-soluble, which has attracted extensive research attention.^21–23^ CDs can be anchored onto nanomaterial surface of nanomaterials to render inherently hydrophobic nanomaterials dispersible in aqueous solution.^24,25^ Additionally, the hydrophobic inner cavity of CDs can interact with specific guest molecules *via* host–guest interactions, enabling efficient drug encapsulation.^26^ The host–guest complexes formed by CDs and drugs typically exhibit 2 : 1 or 1 : 1 stoichiometries, a feature that allows one or more CD molecules to encapsulate a single drug molecule.^27,28^ Among the reported CDs/guest systems, the combination of β-CD and adamantane is the most well-established. The host–guest interaction between β-CD and adamantane is extremely strong, with an association constant of 10^5^ M^−1^, which typically forms 1 : 1 host–guest complexes.^29,30^ Therefore, engineering host–guest complexation between porphyrin-based photosensitizers (guests) and CDs offers a strategy to suppress intermolecular interactions between photosensitizers, alleviate aggregation-induced quenching, and ultimately boost PDT efficacy.

Herein, we propose a host–guest complexation-based isolation strategy to inhibit photosensitizer aggregation and enhance photodynamic killing efficacy. Specifically, we synthesized a supramolecular nano-platform Cu_2−*x*_Se-CD, featuring a core of ultra-small cuprous selenide nanoparticles (Cu_2−*x*_Se NPs) surface-modified with mercapto-β-cyclodextrin (HS-β-CD). Then Cu_2−*x*_Se-CD was employed to load adamantane-functionalized 5-(4-hydroxyphenyl)-10,15,20-triphenylporphyrin (Ada-TPP), yielding the supramolecular nano-photosensitizer Cu_2−*x*_Se-CD-TPP (Scheme 1). Cu_2−*x*_Se-CD-TPP exhibited an ultrasmall size of ∼4–6 nm and excellent aqueous dispersibility, and compared to free Ada-TPP, Cu_2−*x*_Se-CD-TPP showed an 84.8% increase of singlet oxygen yield under laser irradiation. Moreover, the supramolecular nano-photosensitizer exhibited excellent photoacoustic imaging capabilities. Ultimately, Cu_2−*x*_Se-CD-TPP exhibited an excellent therapeutic efficacy of PDT. This study not only provides a new strategy to address the photosensitizer aggregation challenge but also expands the application of host–guest chemistry in tumor treatment.

**Scheme 1:**
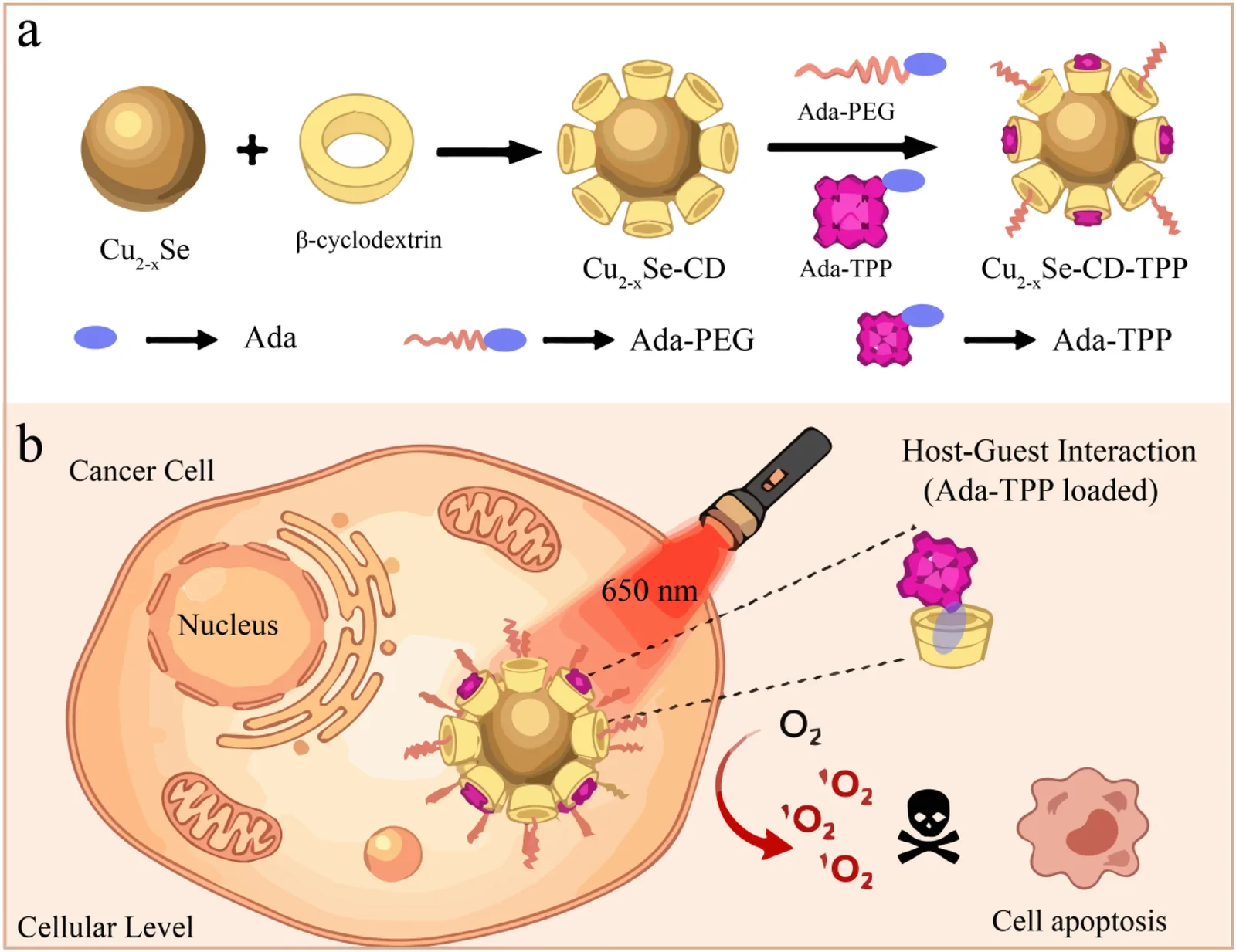
(a) Synthetic route of Cu_2−*x*_Se-CD-TPP; (b) the anti-tumor process of Cu_2−*x*_Se-CD-TPP.

## 2. Results and discussion

### 2.1 Construction of ultra-small supramolecular loading nanoplatform (Cu_2−*x*_Se-CD)

The fabrication of the ultra-small supramolecular loading nanoplatform Cu_2−*x*_Se-CD was achieved *via* two key steps. Briefly, non-stoichiometric ultra-small Cu_2−*x*_Se nanoparticles were synthesized through NaBH_4_-mediated reduction of Se powder, followed by the addition of CuCl_2_.^31,32^ As shown in Fig. 1a, the X-ray diffraction (XRD) pattern of the as-synthesized Cu_2−*x*_Se matches well with the standard reference (PDF no. 06-0680). A transmission electron microscopy (TEM) image revealed that the synthesized Cu_2−*x*_Se nanoparticles have an ultra-small average core size of 2–3 nm (Fig. 1b).

**Fig. 1:**
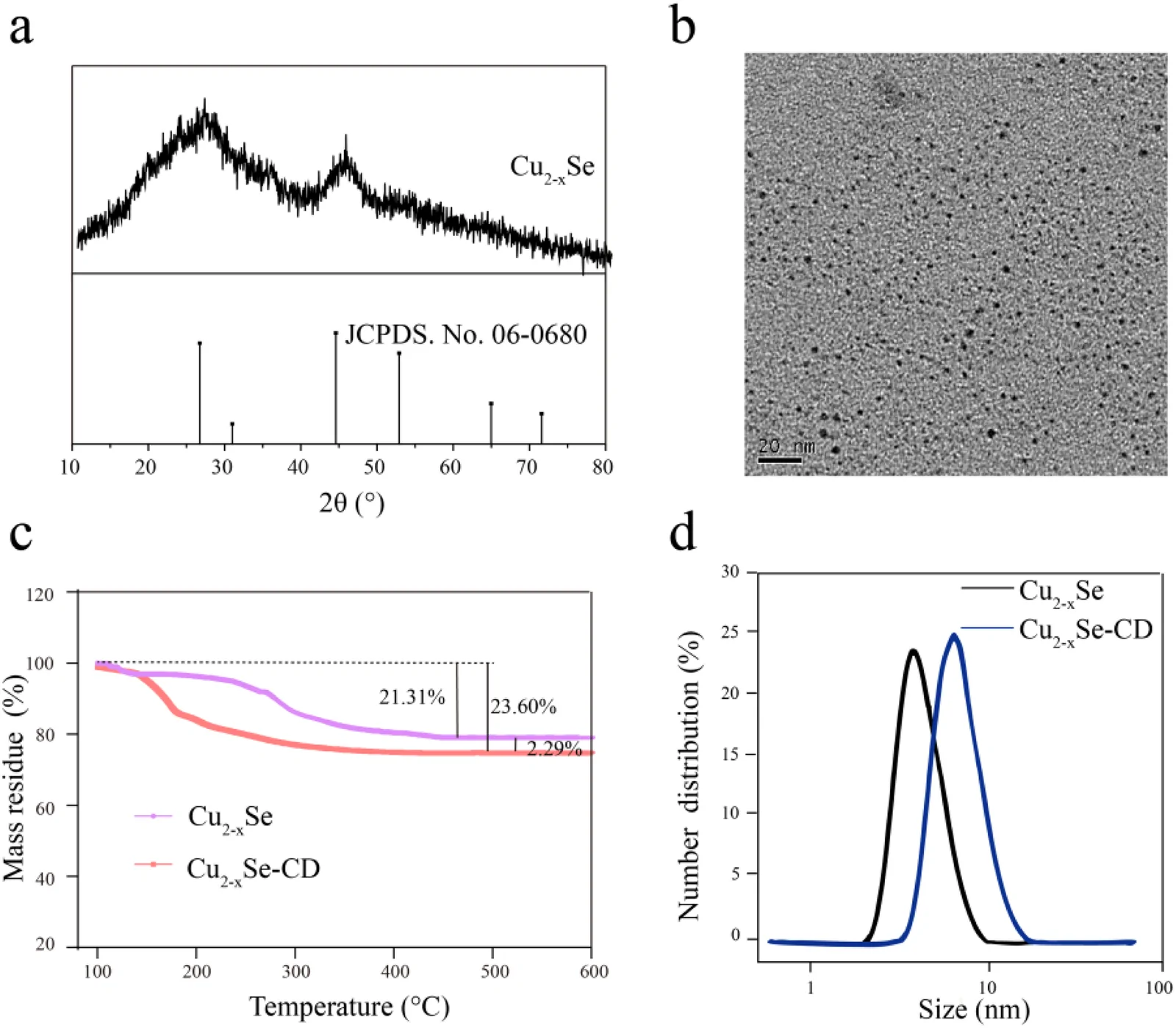
The construction of Cu_2−*x*_Se-CD nano-platform. (a) The XRD spectra of Cu_2−*x*_Se. (b) The TEM image of Cu_2−*x*_Se. (c) The TGA curves of Cu_2−*x*_Se and Cu_2−*x*_Se-CD. (d) The DLS size of Cu_2−*x*_Se and Cu_2−*x*_Se-CD.

Subsequently, the as-prepared Cu_2−*x*_Se nanoparticles were functionalized with mercapto-β-cyclodextrin (HS-β-CD) *via* the strong coordination interaction between thiol groups and copper ions, yielding the final Cu_2−*x*_Se-CD nanoplatform. As shown in Fig. 1c, thermogravimetric analysis (TGA) revealed that Cu_2−*x*_Se-CD exhibited substantially higher weight loss than unmodified Cu_2−*x*_Se over the temperature range of 200–400 °C. This weight loss is typically attributed to the thermal decomposition of the organic component (HS-β-CD). These results confirm the successful grafting of HS-β-CD onto the surface of Cu_2−*x*_Se nanoparticles, and this conclusion is further supported by the observed changes in dynamic light scattering (DLS) size and zeta potential (Fig. 1d and S1). Collectively, the above characterization results verify the successful preparation of the Cu_2−*x*_Se-CD nanoplatform.

### 2.2 Preparation of ultra-small supramolecular nano-photosensitizers (Cu_2−*x*_Se-CD-TPP)

To obtain ultra-small supramolecular nano-photosensitizers (Cu_2−*x*_Se-CD-TPP), adamantane-functionalized 5-(4-hydroxyphenyl)-10,15,20-triphenylporphyrin (Ada-TPP) was synthesized following previously reported procedures.^33,34^ As shown in Fig. 2a, the ^1^H NMR spectrum of purified Ada-TPP displays characteristic resonances corresponding to the phenylporphyrin moiety: signals at 7.0–9.0 ppm and −2.8 ppm, as well as the characteristic adamantane peaks at 1.5–2.0 ppm. The integral ratio of the two sets of signals was calculated to be 1 : 1, confirming the successful synthesis of Ada-TPP. Subsequently, the host–guest complexation between HS-CD and Ada-TPP was systematically investigated. Obvious chemical-shift perturbations of adamantane protons were detected upon mixing HS-CD with Ada-TPP (Fig. 2b), which is indicative of an altered local chemical microenvironment driven by the insertion of the adamantane group into the cyclodextrin cavity. Fluorescence titration experiments revealed concentration-dependent fluorescence enhancement of Ada-TPP with gradual addition of HS-CD (Fig. 2c). Non-linear regression fitting based on a 1 : 1 host–guest binding model yielded an association constant of *K*_*a*_= (2.48 ± 0.27) ×10^5^ M^−1^ (*R*^2^ = 0.988, Fig. S2), further corroborating the efficient host–guest interaction between HS-CD and Ada-TPP, indicating Cu_2−*x*_Se-CD could load Ada-TPP to prepare Cu_2−*x*_Se-CD-TPP.

**Fig. 2:**
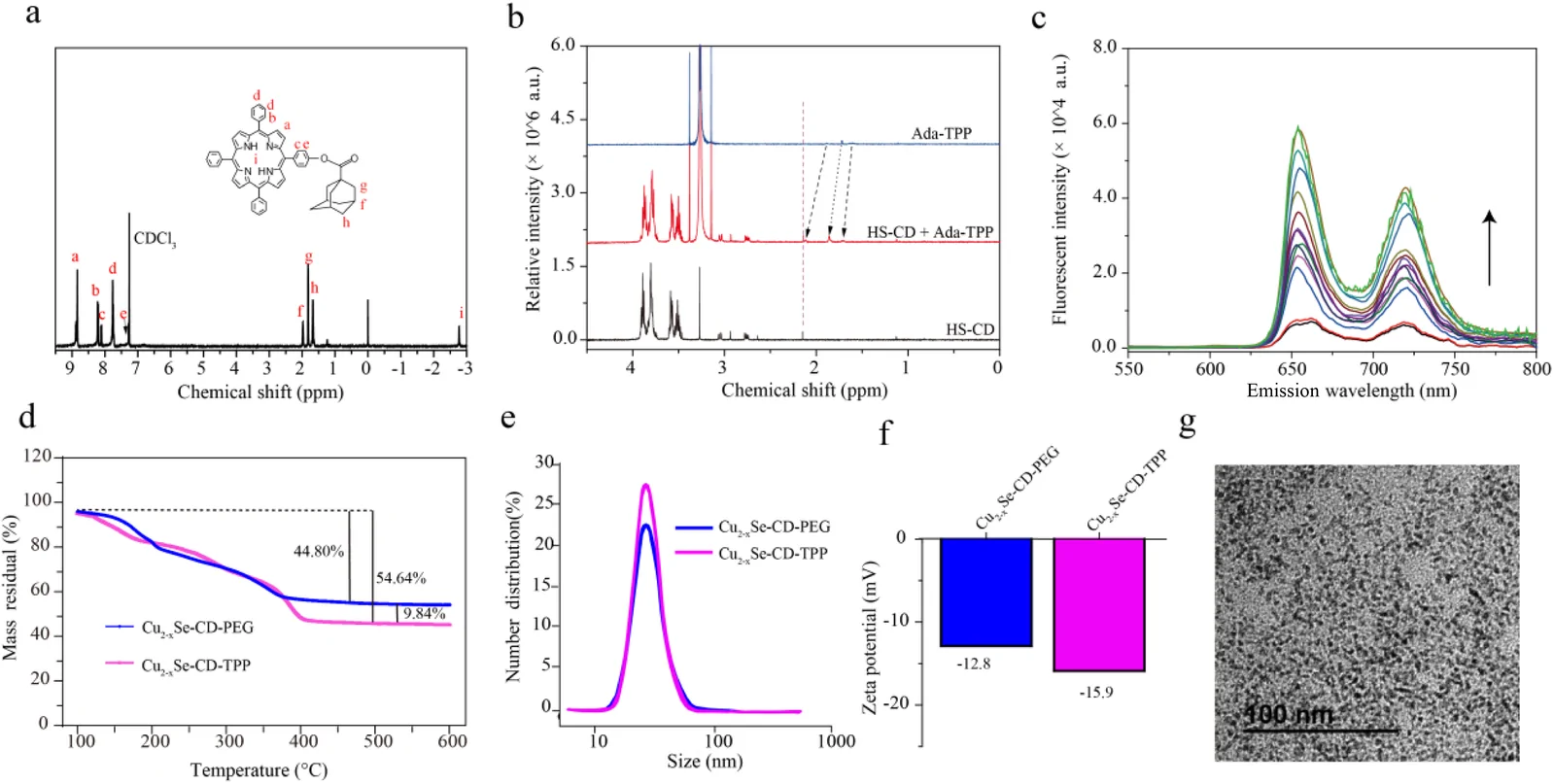
The preparation of Cu_2−*x*_Se-CD-TPP nano-photosensitizers (a) the ^1^H NMR spectrum of Ada-TPP. (b) ^1^H NMR spectra of free HS-CD, free Ada-TPP, and HS-CD + Ada-TPP mixture. (c) Fluorescence spectra of Ada-TPP (1.0 × 10^−5^ M) in aqueous solution with different concentrations of HS-CD: 0, 0.2, 0.4, 0.6, 0.8, 1.5, 3.0, 4.0, 6.0, 8.0, 10.0, and 12.0 × 10^−5^ M. (d) The TGA curves of Cu_2−*x*_Se-CD-PEG and Cu_2−*x*_Se-CD-TPP. (e) The DLS size of Cu_2−*x*_Se-CD-PEG and Cu_2−*x*_Se-CD-TPP. (f) The zeta potential of Cu_2−*x*_Se-CD-PEG and Cu_2−*x*_Se-CD-TPP. (g) The TEM image of Cu_2−*x*_Se-CD-TPP.

Then, the experiment of Cu_2−*x*_Se-CD loading Ada-TPP was carried out, and to enhance the dispersibility and biocompatibility of nanomaterial, a synthesized adamantane-functionalized polyethylene glycol (Ada-PEG) was added to the system (Fig. S3). As shown in Fig. 2d, Cu_2−*x*_Se-CD-PEG and Cu_2−*x*_Se-CD-TPP exhibited total weight losses of 44.80% and 54.64%, respectively. The increased organic weight loss (9.84%) confirms that Ada-TPP was successfully loaded onto the surface of Cu_2−*x*_Se-CD to obtain the ultra-small supramolecular nano-photosensitizers Cu_2-*x*_Se-CD-TPP. To further validate the successful preparation of Cu_2−*x*_Se-CD-TPP, we also measured the hydrodynamic diameter and zeta potential of both products. As illustrated in Fig. 2e, f and S4a, b, Cu_2−*x*_Se-CD-TPP exhibited favorable stability, while showing distinct physicochemical changes relative to Cu_2−*x*_Se-CD, confirming successful Ada-TPP loading. Meanwhile, TEM imaging of Cu_2−*x*_Se-CD-TPP (Fig. 2g) revealed a particle size of 4–6 nm, which was significantly larger than that of Cu_2−*x*_Se-CD. Additionally, X-ray diffraction (XRD) patterns verified that the as-synthesized Cu_2−*x*_Se-CD-TPP was highly consistent with the standard reference (PDF no. 06-0680), and its crystal structure remained stable compared with pure Cu_2−*x*_Se (Fig. S5). The Ada-TPP loading content was determined to be 10.39 ± 0.42 wt% from the standard curve built upon its Soret-band absorbance at 414 nm (Fig. S6a and b), which is in good agreement with the weight-loss value obtained from TGA measurements. Collectively, these results confirm the successful synthesis of ultra-small supramolecular nano-photosensitizers Cu_2−*x*_Se-CD-TPP.

### 2.3 Evaluation of Ada-TPP aggregation in Cu_2−*x*_Se-CD-TPP

To verify that the host–guest complexation between HS-CD and Ada-TPP could suppress porphyrin aggregation, a series of spectroscopic experiments were performed. As shown in Fig. 3a, Ada-TPP and OH-TPP exhibited a sharp Soret band in methanol, but in aqueous solution, obvious broadening and decreased absorbance of the Soret band were observed, indicating severe aggregation of Ada-TPP and OH-TPP in water. In addition, consistent with aggregation-induced quenching, Ada-TPP and OH-TPP in water displayed heavily suppressed fluorescence emission (Fig. S7). However, it is obvious that Cu_2−*x*_Se-CD-TPP obtained *via* host–guest assembly exhibited well-defined sharp Soret band (Fig. 3b) and Cu_2−*x*_Se-CD-TPP displayed strong fluorescence emission (Fig. 3c). These observations demonstrate that the host–guest complexation between HS-CD and Ada-TPP effectively alleviated porphyrin aggregation.

**Fig. 3:**
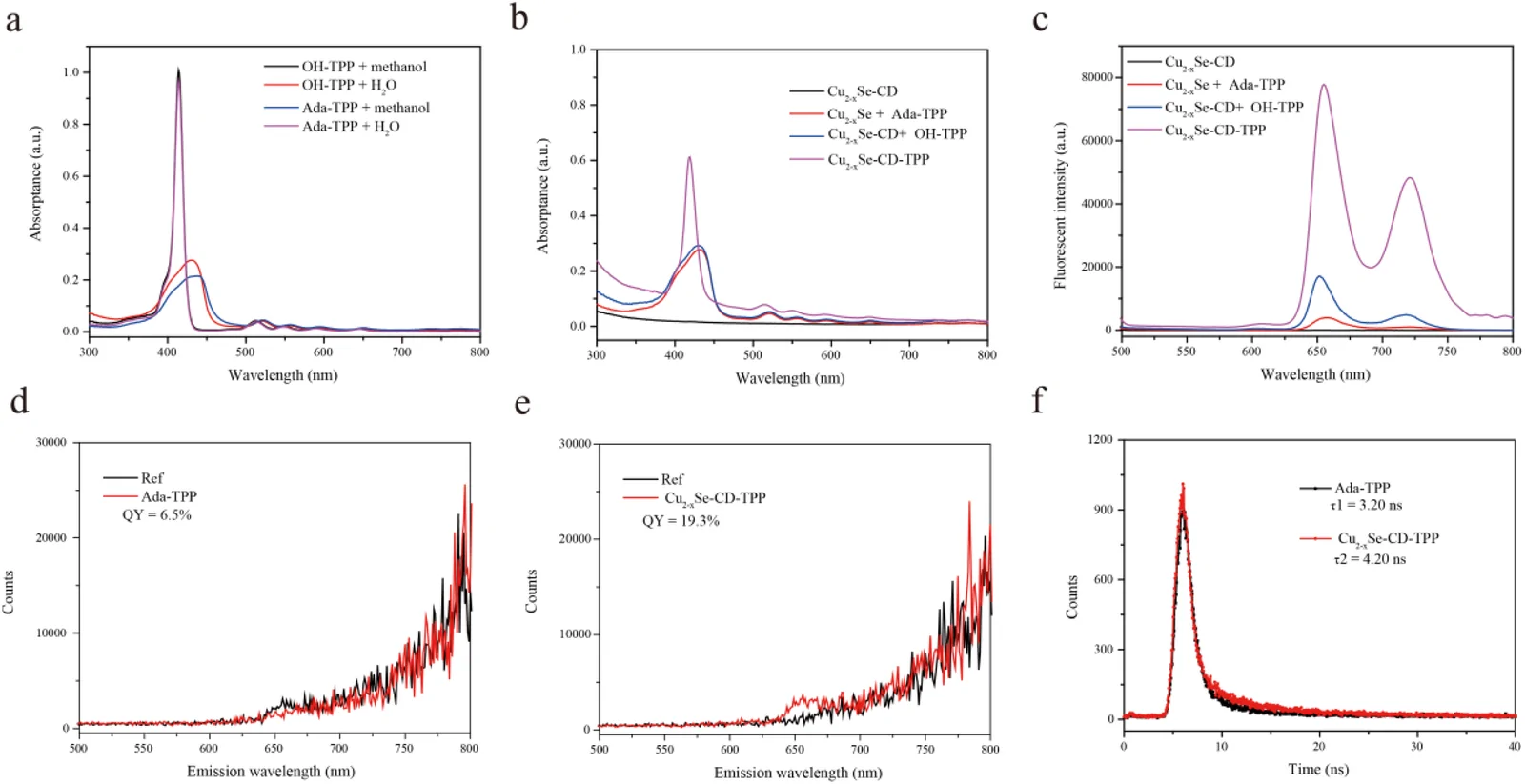
Spectroscopic characterizations of Ada-TPP and Cu_2−*x*_Se-CD-TPP. (a) UV-Vis absorption spectra of Ada-TPP in water and methanol. (b) UV-vis absorption spectra of Cu_2−*x*_Se-CD, Cu_2−*x*_Se + TPP, Cu_2−*x*_Se + OH-TPP and Cu_2−*x*_Se-CD-TPP. (c) Fluorescence emission spectra of different nanocomposites. (d and e) Absolute fluorescence quantum yields of Ada-TPP and Cu_2−*x*_Se-CD-TPP. (f) Fluorescence lifetime decay curves of Cu_2−*x*_Se-CD-TPP and free Ada-TPP measured by an integrating sphere.

To further verify this point, the time-resolved fluorescence decay and absolute fluorescence quantum yields were also measured. As shown in Fig. 3d and e, free aqueous Ada-TPP possessed a low quantum yield of 6.5%, whereas Cu_2−*x*_Se-CD-TPP afforded a much higher quantum yield of 19.3%. In addition, compared with the fluorescence lifetime of Ada-TPP (3.20 ns), that in Cu_2−*x*_Se-CD-TPP (4.2 ns) has been significantly prolonged. All these results indicated that cyclodextrin-adamantane host–guest interaction suppressed non-radiative decay pathways originating from porphyrin intermolecular aggregation.

### 2.4 Evaluation of ROS generation and photoacoustic imaging capabilities of Cu_2−*x*_Se-CD-TPP

To evaluate the singlet oxygen (^1^O_2_) generation capability of Cu_2−*x*_Se-CD-TPP, a 1,3-diphenylbenzofuran (DPBF) degradation assay was performed. As illustrated in Fig. 4a and S8, negligible DPBF degradation was detected for the light-only or Cu_2−*x*_Se-CD + light groups. By contrast, both the Ada-TPP and Cu_2−*x*_Se-CD-TPP groups exhibited pronounced DPBF degradation upon light exposure. Notably, the Cu_2−*x*_Se-CD-TPP group displayed substantially faster degradation kinetics than the Ada-TPP group (Fig. 4b and c). These results confirm that both Cu_2−*x*_Se-CD-TPP and Ada-TPP can generate ^1^O_2_ under light irradiation, and Cu_2−*x*_Se-CD-TPP affords a higher ^1^O_2_ yield than free Ada-TPP. Furthermore, by comparing the DPBF degradation slopes, we obtained a slope of −0.7317 for Ada-TPP and −1.3220 for Cu_2−*x*_Se-CD-TPP (Fig. 4d), corresponding to an 84.8% increase in ^1^O_2_ yield for Cu_2−*x*_Se-CD-TPP relative to the free photosensitizer control. This improvement in ^1^O_2_ production is ascribed to the spatial separation of porphyrin photosensitizers on the Cu_2−*x*_Se-CD ultra-small supramolecular nanoplatform mediated by cyclodextrin, which effectively alleviates aggregation-caused quenching (ACQ).

**Fig. 4:**
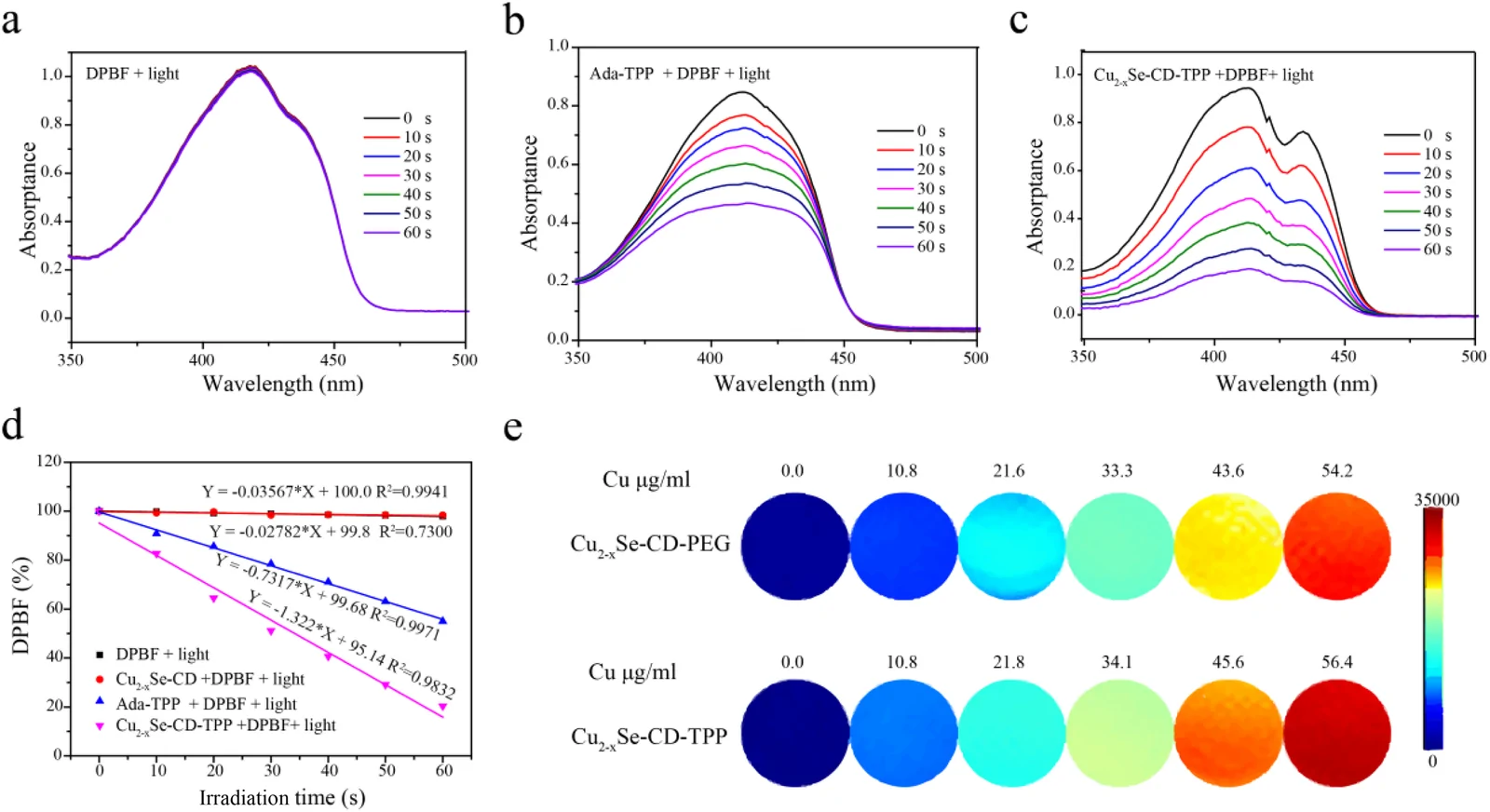
The ROS generation and photoacoustic imaging capabilities of Cu_2−*x*_Se-CD-TPP *in vitro*. (a–c) The UV-Vis absorptance spectra of DPBF in different groups with light irradiation (blank, Ada-TPP and Cu_2−*x*_Se-CD-TPP). (d) The relative UV-Vis absorptance of DPBF at 420 nm with light irradiation time (blank, Cu_2−*x*_Se-CD, Ada-TPP and Cu_2−*x*_Se-CD-TPP). (e) To investigate the photoacoustic imaging (PAI) properties of Cu_2−*x*_Se-CD-PEG and Cu_2−*x*_Se-CD-TPP at various concentrations under 808 nm laser irradiation, as well as the relationship between their concentrations and PAI performance.

Additionally, the photoacoustic (PA) imaging performance of Cu_2−*x*_Se-CD-TPP was evaluated. As illustrated in Fig. 4e, the PA signals of Cu_2−*x*_Se-CD-PEG and Cu_2−*x*_Se-CD-TPP at 808 nm were clearly enhanced with increasing concentrations. Further linear regression analysis (Fig. S9) demonstrated a strong linear correlation between the PA signal intensity and concentration for both samples. Collectively, these findings indicate that Cu_2−*x*_Se-CD-TPP can serve as an effective PA imaging agent for real-time monitoring of its *in vivo* distribution, thereby providing reliable guidance for subsequent PDT.

### 2.5 Evaluation of the cell-killing performance of Cu_2−*x*_Se-CD-TPP

Firstly, cellular internalization of Cu_2−*x*_Se-CD-TPP by 4T1 cells was verified *via* confocal laser scanning microscopy (CLSM). As shown in Fig. 5a, porphyrin red fluorescence was evident within 4T1 cells, with the signal intensity increasing as incubation time prolonged. Together, these observations confirm that 4T1 cells internalize Cu_2−*x*_Se-CD-TPP in a time-dependent manner. Following confirmation of cellular internalization, intracellular reactive oxygen species (ROS) generation by Cu_2−*x*_Se-CD-TPP under light irradiation was assessed using the ROS-sensitive probe DCFH-DA. As shown in Fig. 5b, negligible fluorescence was detected in the groups treated with laser alone or Cu_2−*x*_Se-CD-TPP alone. By comparison, robust green fluorescence was observed in cells treated with Cu_2−*x*_Se-CD-TPP plus light, with the fluorescence intensity significantly higher in the Cu_2−*x*_Se-CD-TPP group than that in the Ada-TPP group. The results revealed that Cu_2−*x*_Se-CD-TPP generated higher levels of ROS than Ada-TPP upon 650 nm laser excitation with equal porphyrin concentrations.

**Fig. 5:**
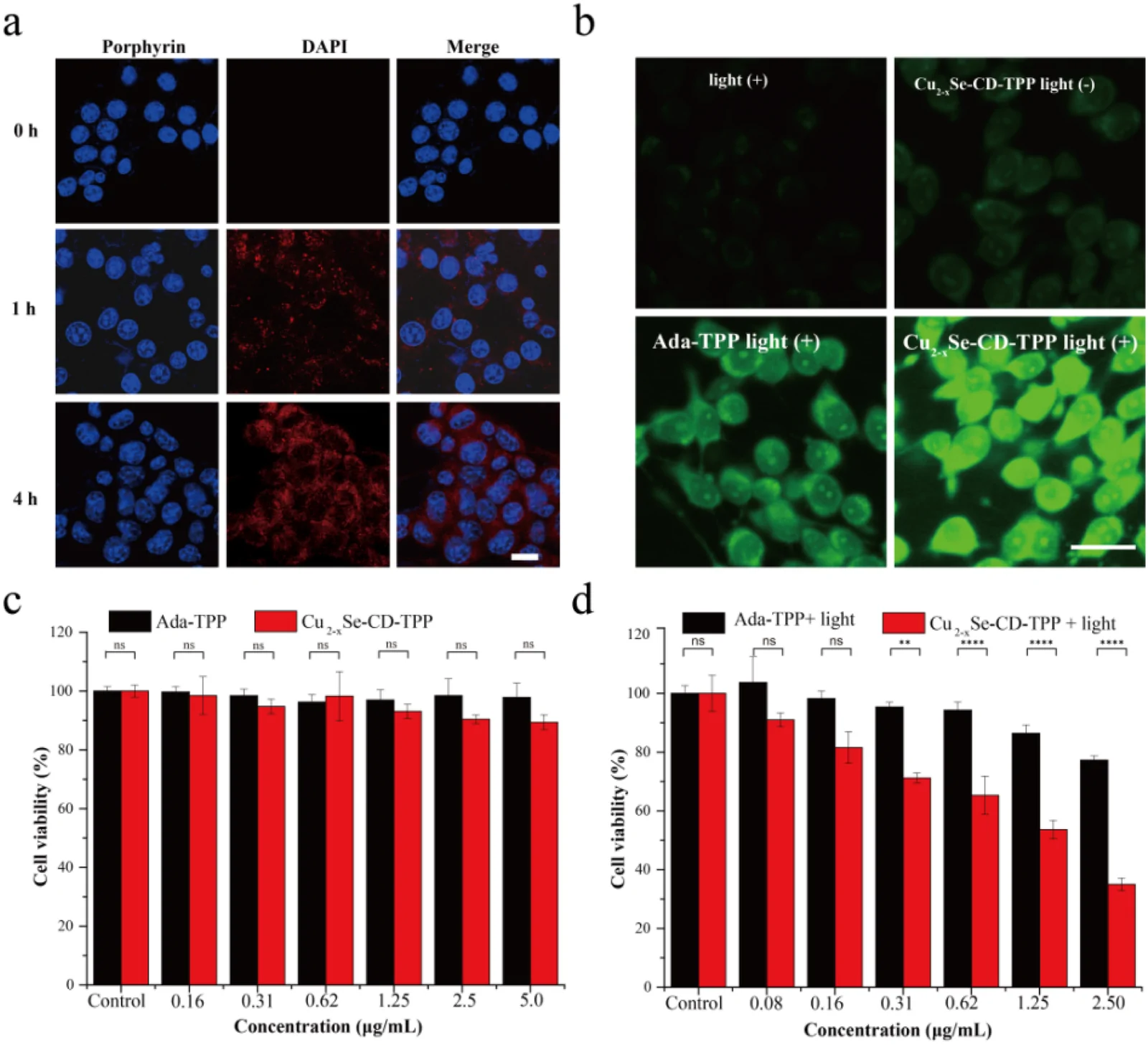
The cytotoxicity of Cu_2−*x*_Se-CD-TPP to 4T1 cells with/without light irradiation. (a) The confocal images of 4T1 cells cultured with Cu_2−*x*_Se-CD-TPP for 1 h and 4 h (scale bar, 20 µm). (b) Confocal images of detection for ROS in 4T1 cells with DCFH (scale bar, 20 µm). (c) The cell viability of Ada-TPP and Cu_2−*x*_Se-CD-TPP to 4T1 cells under dark conditions. (d) The cell viability of Ada-TPP and Cu_2−*x*_Se-CD-TPP to 4T1 cells with light irradiation (650 nm laser, 0.5 W cm^−2^). Data are presented with *n* = 6 technical replicates per group. Statistical comparisons were performed using two-way analysis of variance (ANOVA) followed by Tukey's honestly significant difference (HSD) *post hoc* test for multiple comparisons between concentration groups (ns: not significant, **p < 0.01, ****p < 0.0001).

Building on these ROS generation results, the killing efficacy of Cu_2−*x*_Se-CD-TPP to tumor cells was assessed using the MTT assay. As presented in Fig. 5c and S10, 4T1 cells treated with Ada-TPP or Cu_2−*x*_Se-CD-TPP retained cell viability over 90% at porphyrin concentrations up to 5.0 µg mL^−1^ in the dark. This result confirms the excellent biocompatibility of Cu_2−*x*_Se-CD-TPP without laser irradiation. However, upon 650 nm laser irradiation (0.5 W cm^−2^, 5 min), Cu_2−*x*_Se-CD did not exhibit cytotoxicity at the same copper concentration as Cu_2−*x*_Se-CD-TPP (Fig. S11), and both Ada-TPP and Cu_2−*x*_Se-CD-TPP exerted evident concentration-dependent cytotoxicity toward 4T1 cells (Fig. 5d). Under the same porphyrin content and irradiation parameters, Cu_2−*x*_Se-CD-TPP showed markedly stronger cytotoxicity than Ada-TPP, and produced prominent cell inhibition even at a low porphyrin concentration of 0.16 µg mL^−1^. Collectively, these findings confirm that host–guest interactions suppress the aggregation of porphyrin photosensitizers and further improve their tumor cell killing efficiency.

### 2.6 Evaluation of the anti-tumor effect of Cu_2−*x*_Se-CD-TPP *in vivo*

We next investigated the *in vivo* antitumor efficacy of Cu_2−*x*_Se-CD-TPP using 4T1 xenograft mouse models. As shown in Fig. 6a and b, tumor growth curves and representative photographs of 4T1 tumor-xenografted Balb/c mice after treatments showed rapid tumor growth in the blank, light-only and Cu_2−*x*_Se-CD-TPP-only groups. By contrast, both the Ada-TPP + light and Cu_2−*x*_Se-CD-TPP + light treatments exerted antitumor effects, with the latter producing the most potent tumor inhibition. This trend was further validated by the weights and representative photographs of dissected tumors (Fig. 6c and S12). In addition, mouse body weights remained steady in all groups with no drastic loss (Fig. 6d), demonstrating the good biocompatibility of Cu_2−*x*_Se-CD-TPP.

**Fig. 6:**
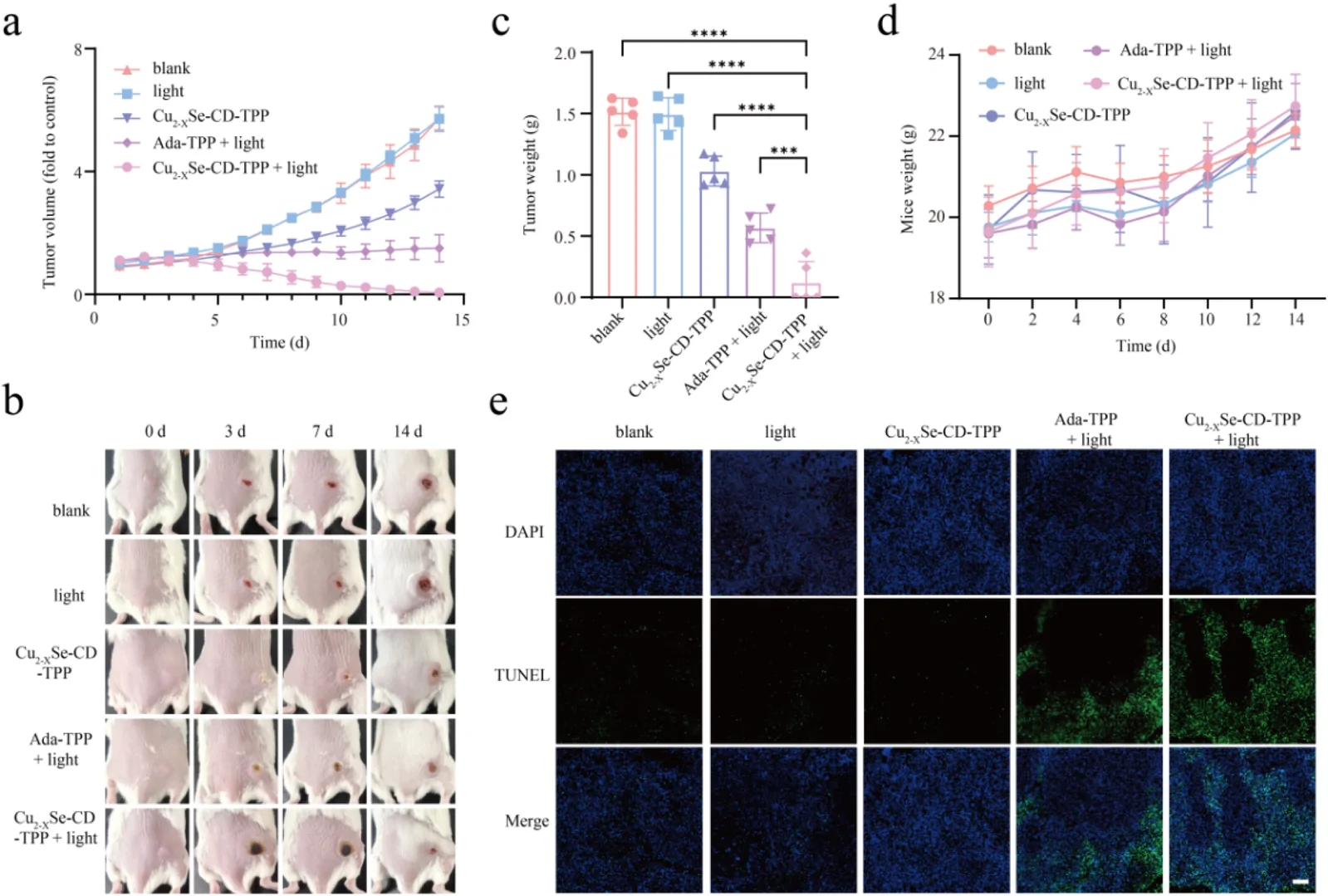
The anti-tumor performance of Cu_2-*x*_Se-CD-TPP *in vivo*. (a) Tumor growth curves of mice after treatments. (b) Representative photographs of 4T1 tumor-xenografted balb/c mice after treatments. (c) Weight of isolated tumors. (d) Body weights of tumor-bearing mice of five groups of mice. (e) TUNEL staining of tumors sections at 48 h after treatments (scale bars: 200 µm). Statistical comparisons were performed using one-way analysis of variance (ANOVA) followed by Tukey's honestly significant difference (HSD) *post hoc* test for multiple comparisons between concentration groups (***p < 0.001, and ****p < 0.0001).

To further prove the efficacy of Cu_2−*x*_Se-CD-TPP under light irradiation, tumor cell apoptosis after treatment was evaluated *via* TUNEL staining (Fig. 6e). Nearly no apoptotic signals were observed in the blank, light-only, and Cu_2−*x*_Se-CD-TPP-only groups. In comparison, positive TUNEL staining was detected in the two combined treatment groups (Ada-TPP + light group and Cu_2−*x*_Se-CD-TPP + light group), and the Cu_2−*x*_Se-CD-TPP + light group exhibited the highest abundance of apoptotic cells. Additionally, we also observed the temperature elevation upon laser irradiation and it was insufficient to induce tumor ablation (Fig. S13 and S14). These results indicate that Cu_2−*x*_Se-CD-TPP upon light irradiation can effectively induce extensive tumor cell apoptosis, accounting for its superior anticancer performance. Overall, these findings proved that host–guest interactions inhibit the aggregation of porphyrin photosensitizers and consequently improve anti-tumor capability.

## 3. Conclusion

In this study, we provided a host–guest complexation-based isolation strategy to suppress porphyrin photosensitizer aggregation and improve the therapeutic efficacy of PDT. Starting from ultra-small Cu_2−*x*_Se nanoparticles and mercapto-β-cyclodextrin, we constructed the Cu_2−*x*_Se-CD supramolecular nanoplatform, which was then loaded with adamantane-functionalized porphyrin photosensitizer to afford the ultra-small Cu_2−*x*_Se-CD-TPP supramolecular nano-photosensitizer with an Ada-TPP loading content of ∼10.39 wt%. The fluorescence titration of the molecular HS-CD/Ada-TPP model system supports a host–guest association, while the spectroscopic characterization of the nanoparticle assembly is consistent with spatial isolation of Ada-TPP, which effectively prevents porphyrin photosensitizers' aggregation. Compared to free photosensitizer, Cu_2−*x*_Se-CD-TPP exhibited an 84.8% increase in ^1^O_2_ production yield and achieved efficient tumor cell killing. In addition, Cu_2−*x*_Se-CD-TPP displayed an excellent anti-tumor performance. Overall, this work not only developed a novel strategy to prevent photosensitizer aggregation, but also develops a promising nanomaterial for high-efficacy PDT.

## Ethical statement

All animal procedures were performed in accordance with the Guidelines for Care and Use of Laboratory Animals of Tongji University and experiments were approved by the Animal Ethics Committee of Tongji University (Approval Number: TJ-HB-LAC-202305).

## Author contributions

Y. W. and C. W., conceived and designed the research. C. L., C. W., and L. Y. performed the related experiments and prepared the nanomaterials. C. L. and C. J. evaluated the functions of nanomaterials *in vitro* and *in vivo*. C. L., and C. W. arranged and analyzed the data. C. L., and C. W. wrote the manuscript. C. L., C. W., and Y. W. revised the paper. All authors contributed to the discussion results and commented on the manuscript.

## Conflicts of interest

The authors declare no competing interests.

## Supplementary Material

RA-OLF-D6RA05237A-s001

## Data Availability

All supporting data generated and analysed in this study are included within the article and the accompanying supplementary information (SI). The raw experimental data that support the findings of this work are available from the corresponding author upon reasonable request. Supplementary information is available. See DOI: https://doi.org/10.1039/d6ra05237a.
