# Supplementary material for: Reducing photosensitizer aggregation via cyclodextrin–adamantane porphyrin host–guest interactions for enhanced photodynamic therapy
Source: RSC Adv. 2026 Oct 27;16(49):66534–42. doi: 10.1039/d6ra05237a (PMC13711117; doi:10.1039/d6ra05237a)
Supplement: Supplementary file 1 [file RA-016-D6RA05237A-s001.pdf]

Supplementary Information (SI) for RSC Advances.
This journal is © The Royal Society of Chemistry

Reducing Photosensitizer Aggregation via Cyclodextrin–Adamantane Porphyrin Host–Guest Interactions for Enhanced Photodynamic Therapy

Chengcheng Liu[a,b], Lijun Yue[b], Chuning Jia[b], Yelin Wu[a,b,*], Chaochao Wang[b,*]

a. School of Medicine, Anhui University of Science and Technology, Huainan 232001, P. R. China.

b. Institute of Hepatobiliary and Pancreatic Surgery, Department of Hepatobiliary and Pancreatic Surgery, Shanghai East Hospital, School of Medicine, Tongji University, Shanghai, 200120, P. R. China.

*E-mail: sk_wuyelin@tongji.edu.cn; wcchao@tongji.edu.cn

Table of Contents

1. Experimental Section

2. Supporting Figures

1. Experimental Section

1.1 Materials

$CuCl_2 \cdot 2H_2O$, selenium powder (Se), sodium borohydride ($NaBH_4$), triethylamine (TEA), mercaptosuccinic acid (MSA), N,N-dimethylformamide (DMF), sodium bicarbonate ($NaHCO_3$), anhydrous sodium sulfate (Na_2SO_4), mercapto-β-cyclodextrin (HS-β-CD), dichloromethane (CH₂Cl_2, SafeDry), 1,3-Diphenylisobenzofuran (DPBF), 1-adamantanecarboxylic acid chloride (Ada-Cl), methoxypolyethylene glycol (m-PEG-OH), and 5-(hydroxyphenyl)-10,15,20-triphenylporphyrin (TPP-OH) were all purchased from Adamas. 3-(4,5-dimethylthiazol-2-yl)-2, 5-diphenyltetrazolium bromide (MTT), 4',6-diamidino-2-phenylindole (DAPI), and 2',7'-dichlorofluorescein diacetate (DCHF-DA) were purchased from Beyotime Biotechnology. All other reagents were obtained from commercial resources and used as received unless otherwise noted.

1.2 Characterization

Dynamic light scattering (DLS) size and distribution, and zeta potential measurements were carried out at Malvern Instruments ZS90. X-ray diffraction (XRD) patterns were gained using a Rigaku D/MAX-2250 V diffractometer. Transmission electron microscopy images were performed on a JEM-F200 transmission electron microscopy working at 200

kV. Confocal laser scanning microscopy (CLSM) images were obtained using a Nikon A1+R-980 confocal microscope. Inductively Coupled Plasma Optical Emission Spectrometer (ICP-OES) were respectively used to measure the concentration Cu.

1.3 Preparation of ultra-small $Cu_{2-x}Se$ nanoparticles

Typically, 100 mL of deionized water was added into a three-necked flask, and the system was deoxygenated by purging with argon (Ar) for 30 min. Subsequently, 79.0 mg (1.00 mmol) of selenium powder was added under continuous magnetic stirring, followed by the addition of 113.6 mg (3.00 mmol) of sodium borohydride ($NaBH_4$) 5 min later. Full reduction of selenium powder was visually confirmed by a characteristic sequential color change: from the initial gray to pink, and finally to a clear colorless solution. Then, an orange-yellow aqueous solution containing 341.0 mg (2.00 mmol) of $CuCl_2{\cdot}H_2O$ and 2.00 g (13.33 mmol) of mercaptosuccinic acid was added dropwise to the reaction mixture. The mixture was stirred continuously for 2 h, with an inert Ar atmosphere maintained throughout the entire process. After reaction completion, the as-synthesized $Cu_{2-x}Se$ nanoparticles were washed via two ultrafiltration steps. Ultrafiltration tubes with a molecular weight cutoff (MWCO) of 300,000 Da were used, and each purification cycle was performed by centrifugation at 4500 rpm for 10 min. After ultrafiltration, brownish-black aqueous dispersion of $Cu_{2-x}Se$ nanoparticles were harvested from the upper fraction of the ultrafiltration tube. The product was stored in a refrigerator, and its copper content was quantified via ICP-OES (3.0 mg/mL).

1.4 Preparation of ultra-small supramolecular loading nanoplatform $Cu_{2-x}Se$-CD

15 mL of the as-prepared $Cu_{2-x}Se$ aqueous dispersion was subjected to two additional purification rounds via ultrafiltration. This process used 300,000 Da molecular weight cutoff (MWCO) ultrafiltration tubes, with each purification cycle performed by centrifugation at 4500 rpm for 10 min. After completion of ultrafiltration, the purified $Cu_{2-x}Se$ nanoparticles were redispersed in 30 mL of deionized water. Subsequently, 225 mg of mercapto-β-cyclodextrin (HS-β-CD) was added to the dispersion, and the mixture was stirred for 24 h to enable surface modification. After the reaction, the as-synthesized $Cu_{2-x}Se$-CD nanoparticles were washed by ultrafiltration with the same specification of ultrafiltration tubes, following the identical centrifugation parameters (4500 rpm, 10 min per cycle). The final $Cu_{2-x}Se$-CD nanoparticles was stored in a refrigerator at 4 °C and its copper content was 3.0 mg/mL.

1.5 Synthesis of adamantane-functionalized 5-(hydroxyphenyl) -10,15,20-phenylporphyrin (Ada-TPP) and its standard curve based on the Soret-band absorbance at 414 nm

0.63 g (1.00 mmol) of 5-(hydroxyphenyl)-10,15,20-triphenylporphyrin (TPP-OH) was dissolved in 15 mL of CH_2Cl_2, and two drops of triethylamine were added into the solution. The reaction mixture was purged with argon gas for 30 min, then cooled to 0 °C in an ice bath. A solution of 1-adamantanecarbonyl chloride (0.30 g, 1.5 mmol) dissolved in 10 mL of CH_2Cl_2 (SafeDry) was added dropwise slowly. The mixture was stirred at 0 °C for 1 h, followed by continued stirring at room temperature for 23 h. Upon completion, the reaction mixture was diluted with 100 mL of CH_2Cl_2, washed successively with aqueous

sodium bicarbonate ($NaHCO_3$) once and brine (aqueous NaCl) three times, then dried over anhydrous sodium sulfate. The mixture was filtered, concentrated under reduced pressure, and purified by silica gel column chromatography using petroleum ether/ethyl acetate (4:1, v/v) as the eluent to afford Ada-TPP as a dark purple solid (0.67 g, 78.8% yield). ^1H NMR (400 MHz, $CDCl_3$): δ 8.83 (d, 8H), 8.21 (m, 6H), 8.12 (d, 2H), 7.79 (m, 9H), 7.29 (d, 2H), 1.98 (m, 3H), 1.82 (d, 6H), 1.68 (m, 6H), -2.78 (s, 2H).

The standard curve of Ada-TPP was constructed via UV-vis absorption spectroscopy. Briefly, a series of Ada-TPP solutions with gradient concentrations (0.50, 0.75, 1.0, 2.0, 4.0, 6.0 and 10 μg mL^{-1}) were prepared. The UV-vis absorption spectra of these Ada-TPP solutions were recorded from 350 to 600 nm. The absorbance values at the Soret band (414 nm) were extracted to establish the linear standard curve. The fitting equation was Y=0.204*X+0.003, with a high correlation coefficient R^2=0.9998.

1.6 Synthesis of adamantane-functionalized polyethylene glycol (Ada-PEG)

4.00 g (2.00 mmol)) of methoxypolyethylene glycol (mPEG-OH) was dissolved in 15 mL of CH_2Cl_2, and two drops of triethylamine were added into the solution. The mixture was purged with argon gas for 30 min, then cooled to 0 °C in an ice bath. A solution of 1-adamantanecarbonyl chloride (0.99 g, 5.00 mmol) dissolved in 10 mL of CH_2Cl_2 was added dropwise slowly. The reaction was stirred at 0 °C for 1 h, then continued at room temperature for 23 h. After completion, the reaction mixture was concentrated under reduced pressure to 2-3 mL. The crude product was precipitated three times with anhydrous diethyl ether, collected, and dried in a vacuum oven to obtain Ada-PEG as a white powdery solid (4.11 g, 92.6% yield). ^1H NMR (400 MHz, $CDCl_3$): δ 3.67 (t, J = 5.0

Hz, 180H), 3.40 (s, 3H), 1.98 (m, 3H), 1.82 (d, 6H), 1.68 (m, 6H).

1.7 Fluorescence titration experiments of Ada-TPP and HS-CD

To investigate association constant between HS-CD and Ada-TPP, fluorescence titration experiments were performed in aqueous solution, which had a constant concentration of Ada-TPP (1.0×10^{-5} M) and varying concentrations of HS-CD (0, 0.2, 0.4, 0.6, 0.8, 1.5, 3.0, 4.0, 6.0, 8.0, 10.0, and 12.0×10^{-5} M). Non-linear regression fitting based on the 1:1 host-guest binding analytical equation afforded an association constant $K_a = (2.48 \pm 0.27) \times 10^5\ M^{-1}$ (R^2=0.988) for HS-CD/Ada-TPP complex. The 1:1 host-guest binding analytical equation used for non-linear fitting is shown below:

$$ΔF=(ΔF_{∞}/[G]_0)(0.5[H]_0 +0.5([G]_0 + 1/K_a) - 0.5([H]_0^2 + (2[H]_0(1/K_a - [G]_0) + (1/Ka + [G]_0)^2)^{0.5})$$

Where ΔF is the change in fluorescence intensity at 650 nm; $ΔF_{∞}$ represents the maximum fluorescence change upon full complex saturation; $[G]_0$ is the fixed initial concentration of the guest Ada-TPP (1.0×10^{-5} M); $[H]_0$ denotes the total added concentration of host (HS-CD).

1.8 Preparation of $Cu_{2-x}Se$-CD-PEG and $Cu_{2-x}Se$-CD-TPP

For the preparation of $Cu_{2-x}Se$-CD-PEG: 30 mg of Ada-PEG was added into 5 mL of $Cu_{2-x}Se$-CD nanoparticles. After 12 h stirring at room temperature, the dispersion was purified by ultrafiltration (ultrafiltration tubes) and washed with deionized water three

times to remove free Ada-PEG, affording $Cu_{2-x}Se$-CD-PEG.

Subsequently, 15 mg Ada-TPP was added into the above $Cu_{2-x}Se$-CD-PEG dispersion, followed by 12 h stirring. The mixture was purified by ultrafiltration and washed three times with deionized water to remove unbound Ada-TPP, yielding the final $Cu_{2-x}Se$-CD-TPP nanocomposite.

The purified $Cu_{2-x}Se$-CD-TPP suspension was diluted with deionized water (100×) to bring absorbance at 414 nm within 0.2-0.8. $Cu_{2-x}Se$-CD dispersion with matched nanoparticle concentration was used as blank to subtract nanoparticle light scattering (Ada-PEG has no absorbance at 414 nm and does not interfere with the UV-vis quantification of Ada-TPP.). The absorbance difference at 414 nm between $Cu_{2-x}Se$-CD-TPP and the blank was used to quantify bound Ada-TPP. The total mass of $Cu_{2-x}Se$-CD-TPP was determined by lyophilizing 2 mL of purified nanoparticle suspension, followed by gravimetric weighing with an analytical balance. The obtained solid mass was then corrected by the dilution factor to calculate the nanoparticle concentration in the original stock suspension. And the final Ada-TPP loading content and the loading efficiency was calculated according to the standard curve of Ada-TPP.

The exact formulas are:

$$\text{Loading content (wt\%)} = \frac{\textit{mass of loaded Ada–TPP}}{\textit{total mass of Cu2–xSe–CD–TPP}} \times 100\%$$

$$\text{Loading efficiency (\%)} = \frac{\textit{mass of loaded Ada–TPP}}{\textit{mass of innitially fed Ada–TPP}} \times 100\%$$

All measurements were conducted in three independent replicates. The loading content (10.39 ± 0.42 wt%) and loading efficiency (45.81 ± 1.76%) are reported as mean ± SD, and these values have been updated in the manuscript.

1.9 Singlet oxygen generation capacity of $Cu_{2-x}Se$-CD-TPP

A 0.20 mL aliquot of $Cu_{2-x}Se$-CD-TPP dispersion was diluted 40-fold with deionized water. Subsequently, 2.00 mL of the diluted solution was transferred to a quartz cuvette, and 20 μL of a 2.00 mg/mL 1,3-diphenylisobenzofuran (DPBF) solution in DMF was

added. The mixture was irradiated with a 650 nm laser for different time intervals (0, 10, 20, 30, 40, 50, 60 s), while the UV-Vis absorption spectra in the range of 300-450 nm were recorded simultaneously. For other groups: DPBF + light, Cu_{2-x}Se-CD + light, and an equivalent amount of Ada-TPP (matching the content in Cu_{2-x}Se-CD-TPP) were prepared, respectively. All groups were subjected to the same laser irradiation protocol, and their UV-Vis absorption spectra at different irradiation times were measured accordingly.

1.10 Photoacoustic imaging performance of Cu_{2-x}Se-CD-TPP and Cu_{2-x}Se-CD-PEG

A series of Cu_{2-x}Se-CD-TPP and Cu_{2-x}Se-CD-PEG with varying copper concentrations (0.0, 10.8, 21.6, 33.6, 43.6, 54.2 μg/mL) were prepared. Each solution was sealed in a plastic tube, with air bubbles removed as thoroughly as possible. The photoacoustic signals at 808 nm were then detected for all samples.

1.11 Cellular uptake of Cu_{2-x}Se-CD-TPP

4T1 cells were cultured in RPMI 1640 medium supplemented with 10% fetal bovine serum (FBS) and 1% penicillin-streptomycin (PS). A total of 2 × 10⁵ cells were seeded in 1 mL confocal dishes and incubated for 24 h. The culture medium was then removed, and fresh RPMI 1640 medium containing Cu_{2-x}Se-CD-TPP was added. The cells were further incubated for 1 h and 4 h, respectively. After incubation, the cells were washed three times with phosphate-buffered saline (PBS), fixed with methanol containing 4',6-diamidino-2-phenylindole (DAPI) for 15 min, and observed under a confocal laser scanning microscope (CLSM). DAPI was excited at 405 nm and emitted at 435 nm;

Ada-TPP was excited at 650 nm and emitted at 720 nm.

1.12 Evaluation of intracellular singlet oxygen generation

4T1 cells (2 × 10⁵) were seeded in 1 mL confocal dishes and cultured for 24 h. The original culture medium was replaced with fresh RPMI 1640 medium containing Cu□□□Se-CD-TPP, and the cells were incubated for another 24 h. Subsequently, the cells were incubated with the reactive oxygen species (ROS) probe DCFH-DA for 0.5 h. The medium was removed, and the cells were washed three times with PBS. The cells were then irradiated with 650 nm laser light for 1 min and observed under a CLSM. DCFH-DA was excited at 488 nm and emitted at 515 nm.

1.13 Dark cytotoxicity of Cu_{2-x}Se-CD-TPP toward 4T1 cells

4T1 cells were seeded into two 96-well microtiter plates at a density of 5×10^3 cells per well and cultured in RPMI 1640 medium for 24 h. The cells were then treated with Cu_{2-x}Se-CD-TPP and Ada-TPP at different concentrations (Ada-TPP concentration in Cu□□□Se-CD-TPP: 0, 0.16, 0.31, 0.62, 1.25, 2.5, 5.0, 10, 20, 40, 80 μg/mL) for 24 h under dark conditions. After treatment, the cells were incubated with PBS containing 0.6 mg/mL MTT for 4 h. The MTT solution was then removed, and 100 μL of dimethyl sulfoxide (DMSO) was added to each well to dissolve the formazan crystals. The absorbance at 490 nm was measured using a microplate reader (Bio-TekELx800, USA).

1.14 Phototoxicity of Cu_{2-x}Se-CD-TPP toward 4T1 cells

4T1 cells were seeded into two 96-well microtiter plates at a density of 5×10^3 cells per well and cultured in RPMI 1640 medium for 24 h. The cells were then treated with

$Cu_{2-x}Se$-CD-TPP and Ada-TPP at different concentrations (Ada-TPP concentration: 0, 0.08, 0.16, 0.31, 0.62, 1.25, 2.5 μg/mL) for 24 h. Following treatment, the cells were irradiated with a 650 nm laser at a power density of 0.5 W/cm^2 for 5 min. The irradiated cells were incubated with fresh culture medium for another 24 h, followed by incubation with PBS containing 0.6 mg/mL MTT for 4 h. The MTT solution was removed, and 100 μL of DMSO was added to each well. The absorbance at 490 nm was finally measured using a microplate reader.

1.15 Anti-tumor effect of $Cu_{2-x}Se$-CD-TPP in 4T1 tumor-xenografted Balb/c mice.

Seven-week-old female Balb/c mice (body weight: ~20 g) were purchased from the Shanghai Laboratory Animal Center, Chinese Academy of Sciences. 4T1 xenograft tumors were established in these mice via subcutaneous (s.c.) injection of 1×10^6 4T1 cells suspended in 100 μL of phosphate-buffered saline (PBS) into the right leg. When tumor volumes reached 70-100 mm, the mice were randomly divided into 5 groups (n = 5 per group) for different therapeutic interventions. Injection volume: 50 μL for each mouse; administration route: single intratumoral injection. The concentration of $Cu_{2-x}Se$-CD-TPP nanoparticle dispersion was 9.62 mg/mL, corresponding to a nanoparticle dose of 0.481 mg per mouse. The loaded Ada-TPP dose per mouse was 0.05 mg (calculated from the drug-loading content of 10.39 wt%), which equals to an Ada-TPP dose of 2.5 mg kg⁻^{1} based on the average body weight of mice. For the free Ada-TPP group, an equivalent molar dose of Ada-TPP was administered intratumorally with the identical injection volume (50 μL). At 12 h after intratumoral (i.t.) injection, mice in the 2nd, 4th, and 5th groups were irradiated with 650 nm light at a power density of 0.50 W/cm for 5 minutes,

and the temperature of $Cu_{2-x}Se$-CD-TPP was monitored. Tumor sizes were measured every day using a digital caliper. Tumor volume (V) was calculated according to the formula: V = L (mm)× W(mm)× W (mm)/2. The pathological tissue sections of tumors were collected in 48 h post-treatment for TUNEL staining.

1.16 Statistical Analysis

All data are presented as mean ± standard deviation (SD) from at least three independent experiments. Statistical comparisons were performed using one-way/two-way analysis of variance (ANOVA) followed by Tukey's post hoc test. A p-value < 0.05 was considered statistically significant ($^*p < 0.05$, $^{**}p < 0.01$, $^{***}p < 0.001$, $^{****}p < 0.0001$).

2. Supporting figures

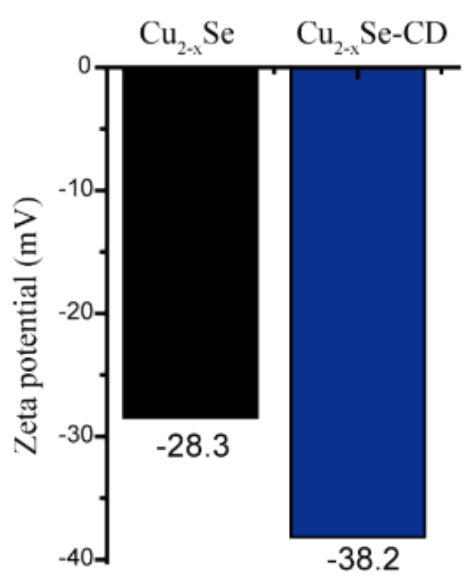

Figure S1. The zeta potential of Cu_2-xSe nanoparticles and Cu_2-xSe-CD nanoparticles.

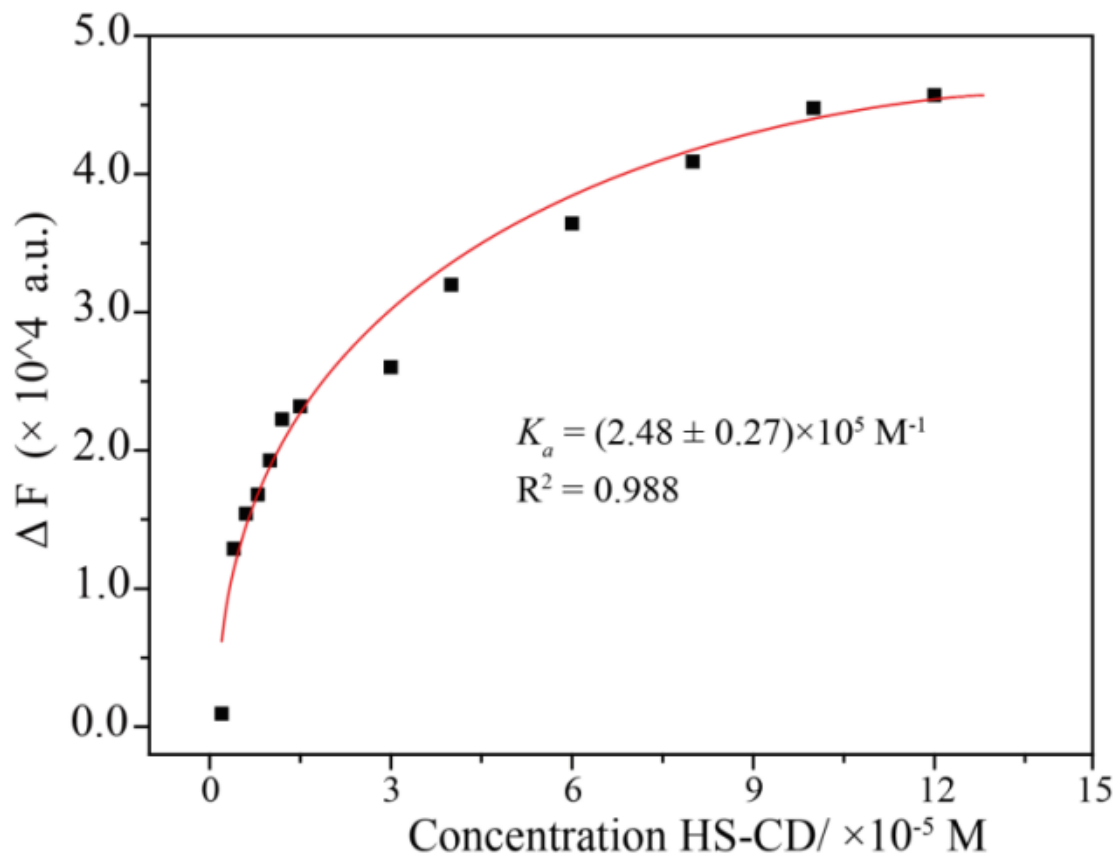

Figure S2. The fluorescence intensity changes upon addition of HS-CD. The red solid line was obtained from the non-linear curve-fitting.

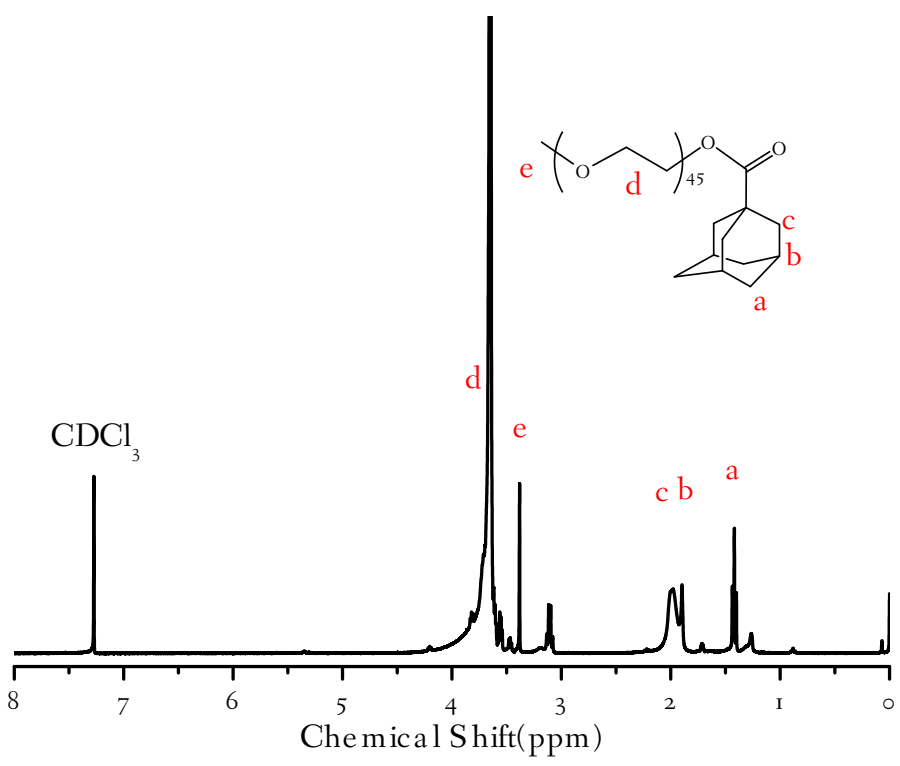

Figure S3. The 1H NMR spectrum of Ada-PEG.

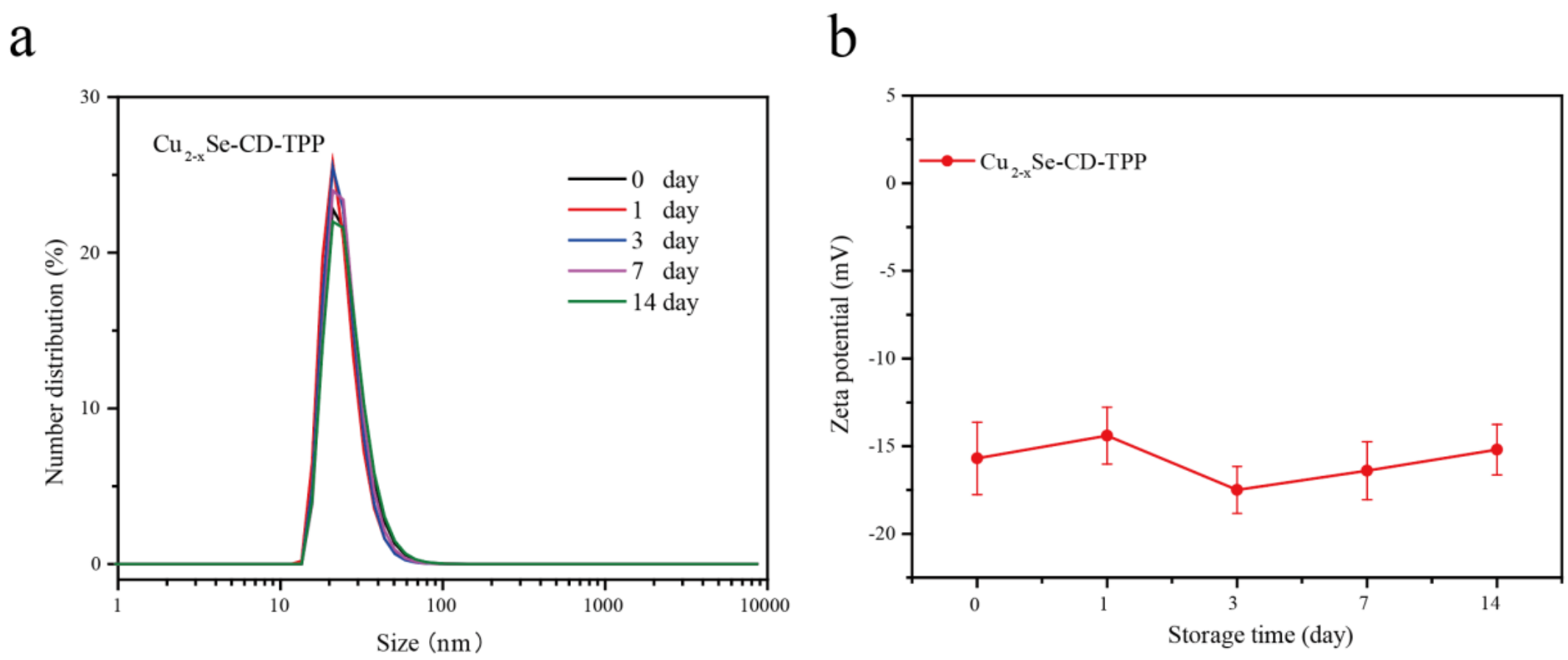

Figure S4. Time-dependent DLS number-size distribution (a) and zeta potential(b) of $Cu_{2-x}Se$-CD-TPP.

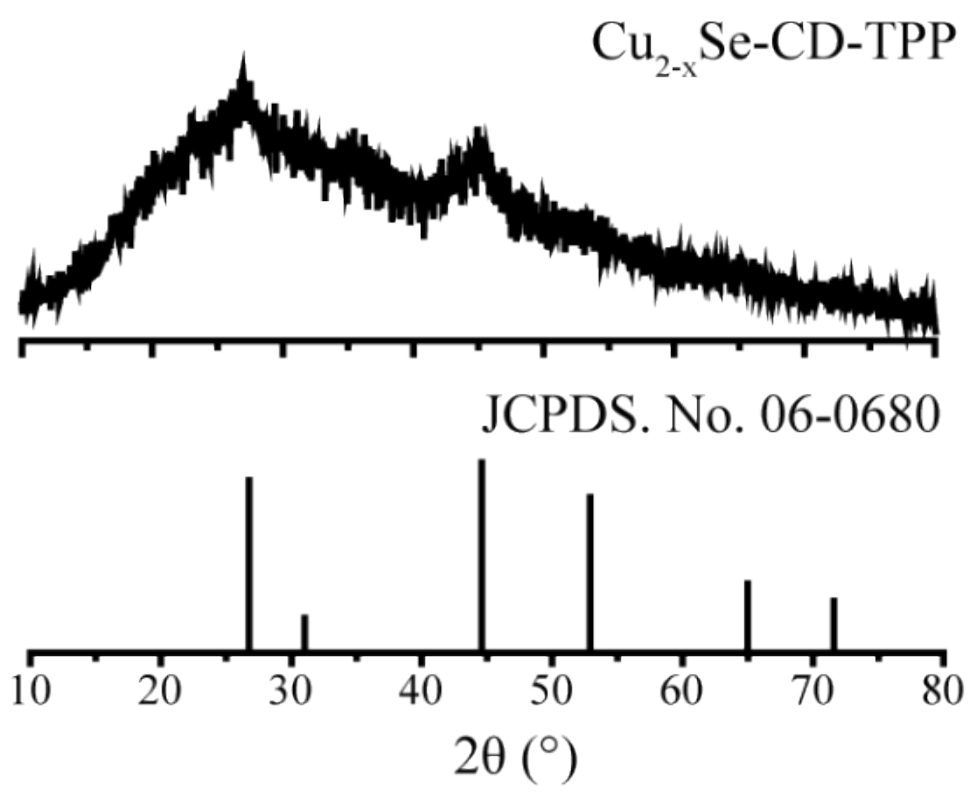

Figure S5 The XRD spectra of $Cu_{2-x}Se$-CD-TPP.

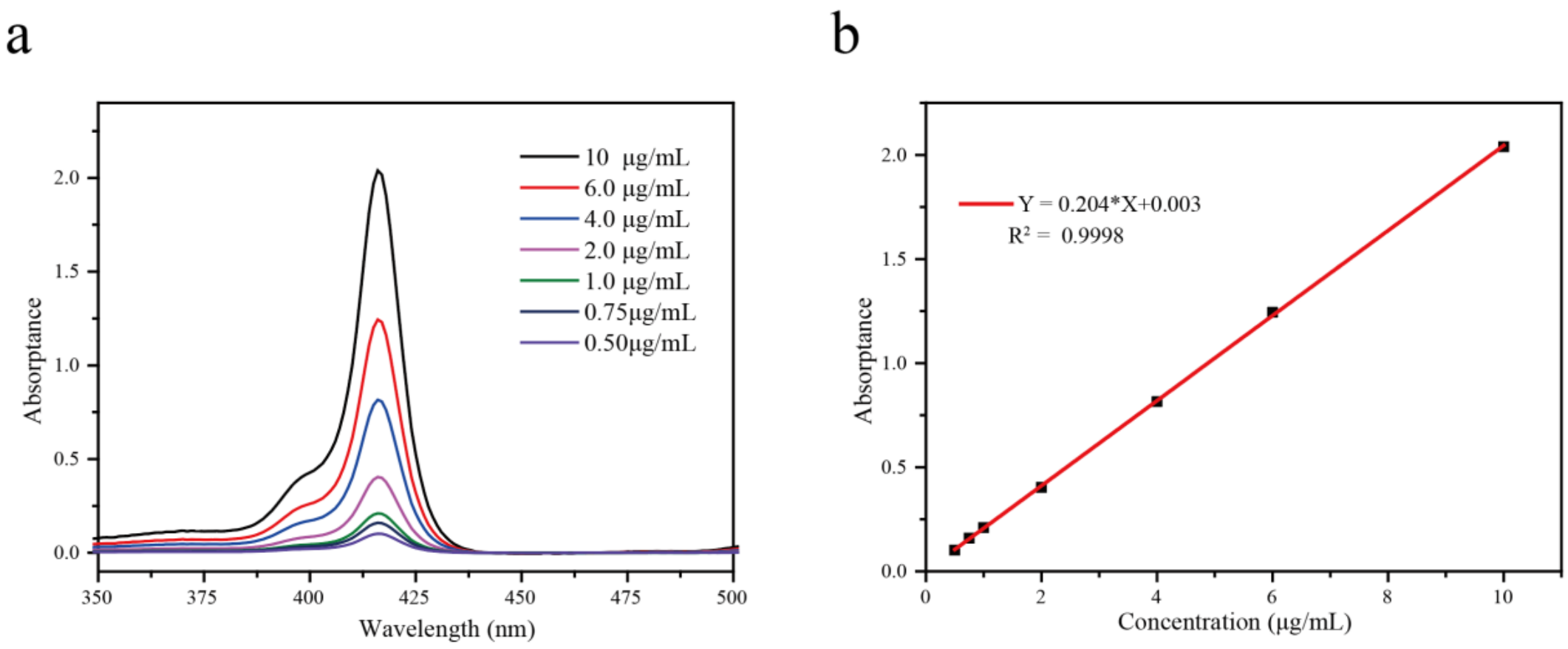

Figure S6. (a) UV-vis absorption spectra of Ada-TPP at different concentrations. (b) Corresponding standard calibration curve of Ada-TPP based on the Soret-band absorbance at 414 nm.

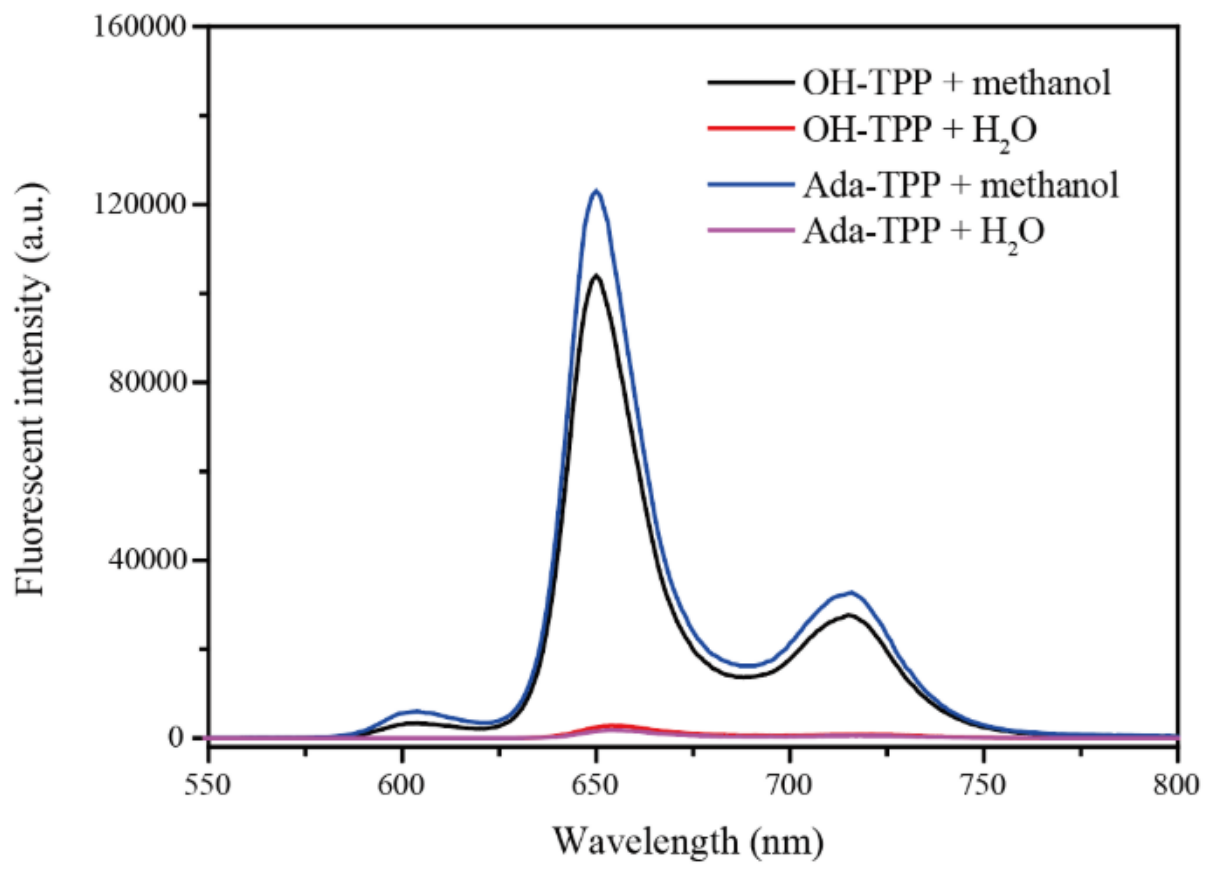

Figure S7. Fluorescence spectra of OH-TPP and Ada-TPP in ethanol and aqueous solutions respectively.

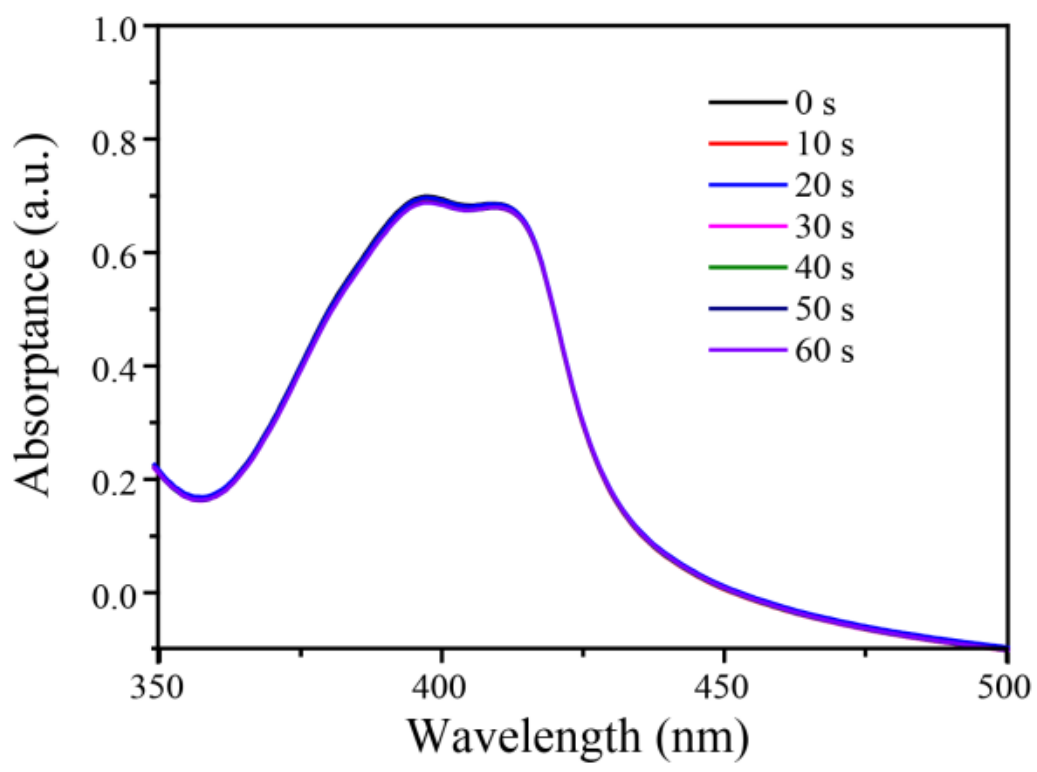

Figure S8. The UV-Vis absorptance spectra of DPBF in $Cu_{2-x}Se$-CD group with irradiation time.

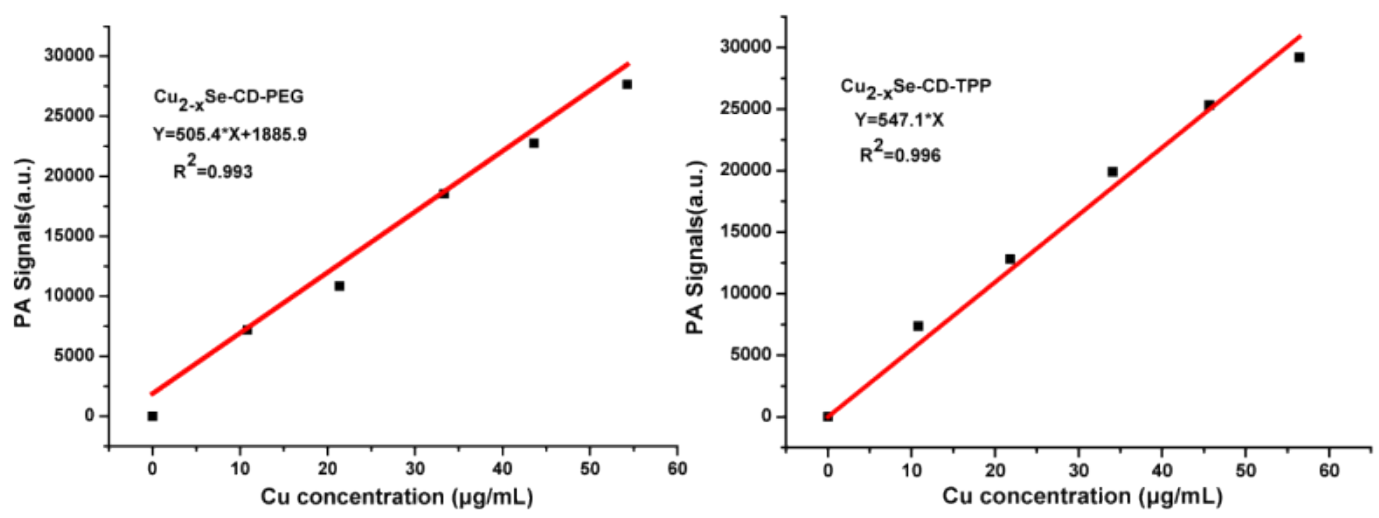

Figure S9. Linear regression analysis of the photoacoustic (PA) signals of $Cu_{2-x}Se$-CD-PEG and $Cu_{2-x}Se$-CD-TPP at the wavelength of 808 nm.

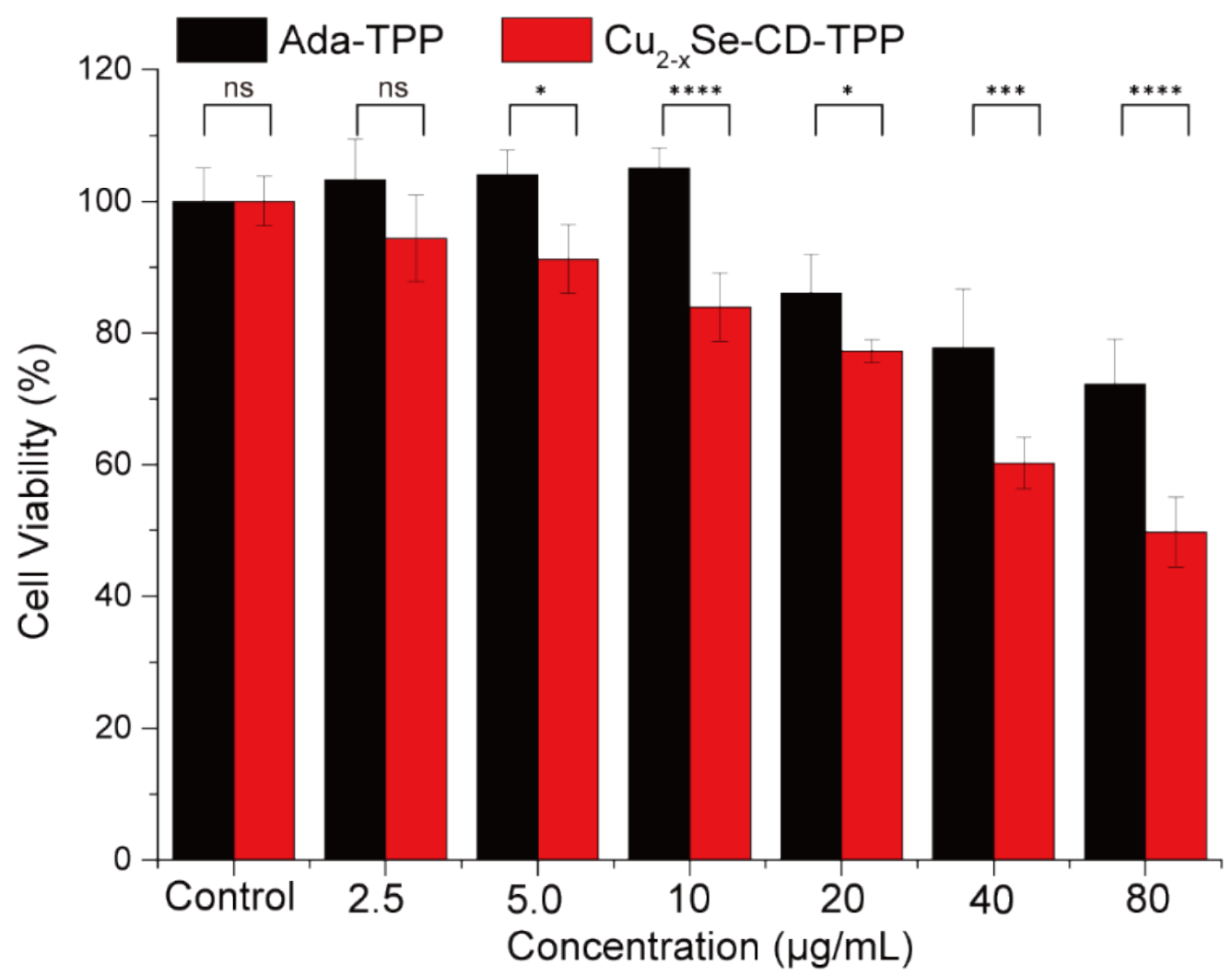

Figure S10. The cell viability of Ada-TPP and $Cu_{2-x}Se$-CD-TPP to 4T1 cells with high concentrations under dark conditions (n=6). Data are presented with n=6 technical replicates per group. Statistical comparisons were performed using two-way analysis of variance (ANOVA) followed by Tukey's honestly significant difference (HSD) post hoc test for multiple comparisons between concentration groups. (*$p<0 .05$, ***$p< 0.001$,and ****$p< 0.001$).

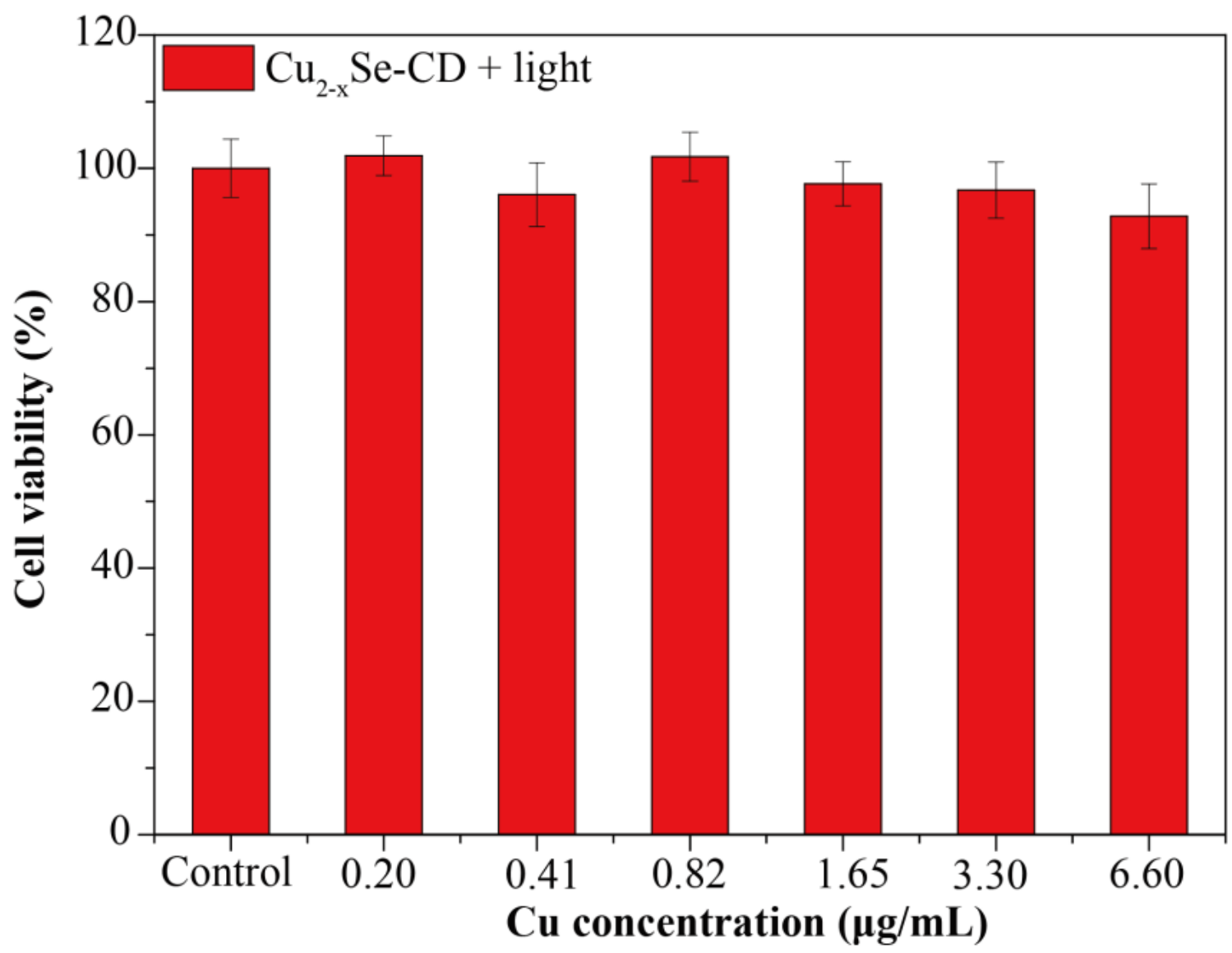

Figure S11. The cell viability of $Cu_{2-x}Se$-CD to 4T1 cells with light irradiation at the same copper concentration as $Cu_{2-x}Se$-CD-TPP (650 nm laser, 0.5 W/cm^2) (n=6) .

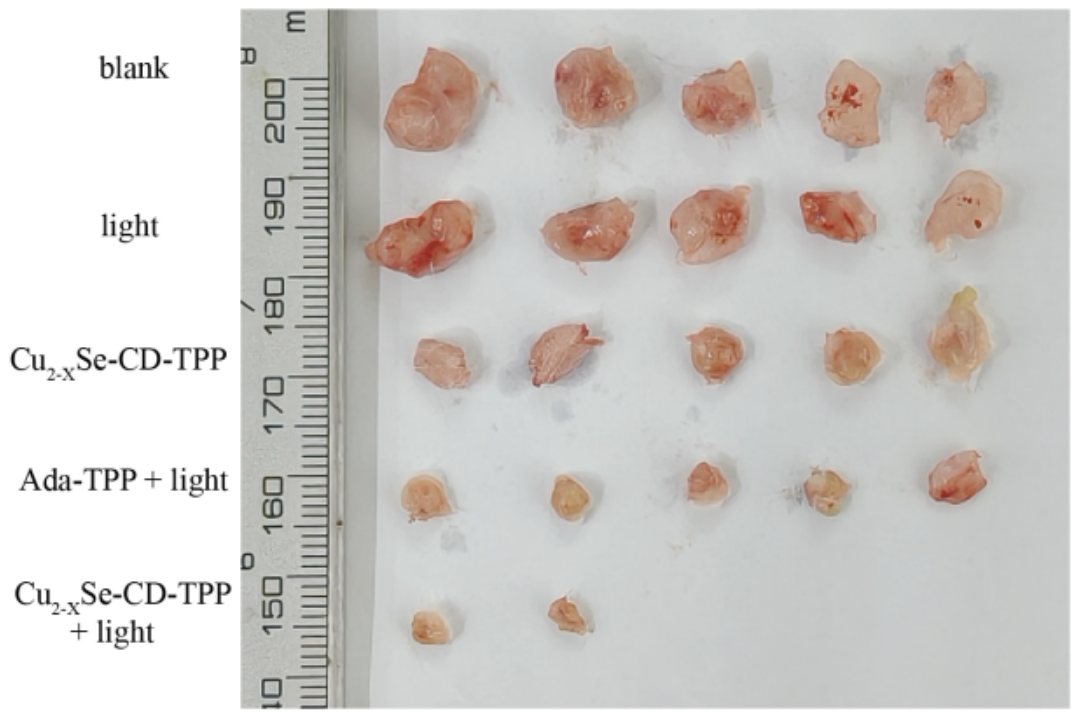

Figure S12. Representative photographs of isolated tumors after treatments.

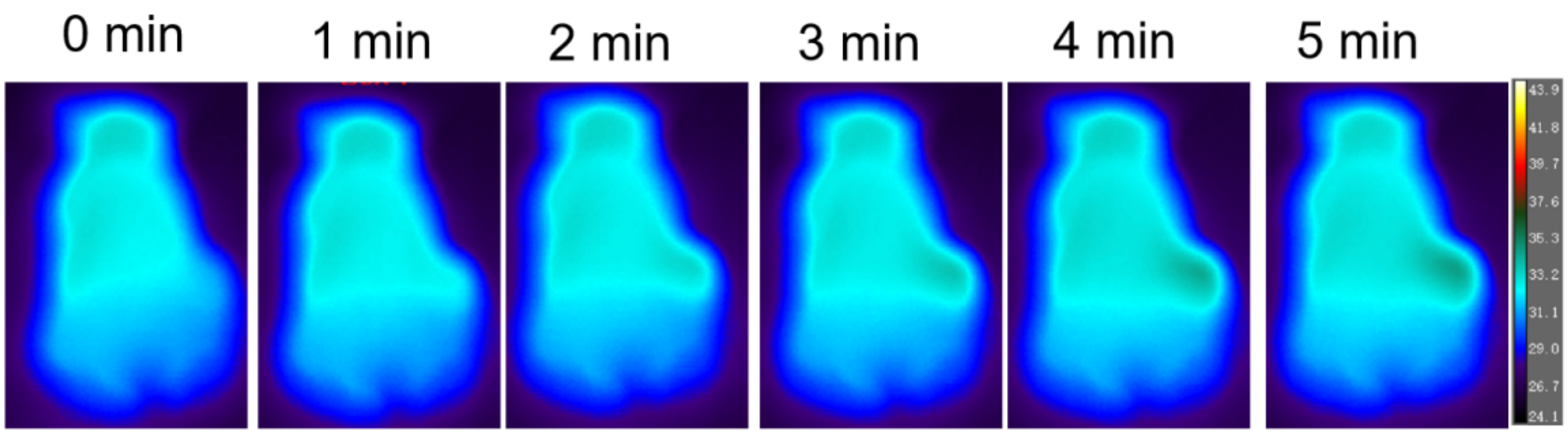

Figure S13. Representative infrared thermal images of tumour-bearing mice (Cu_{2-x}Se-CD-TPP) recorded at 0, 1, 2, 3, 4 and 5 min under 650 nm laser irradiation (0.5 W cm^{-2}).

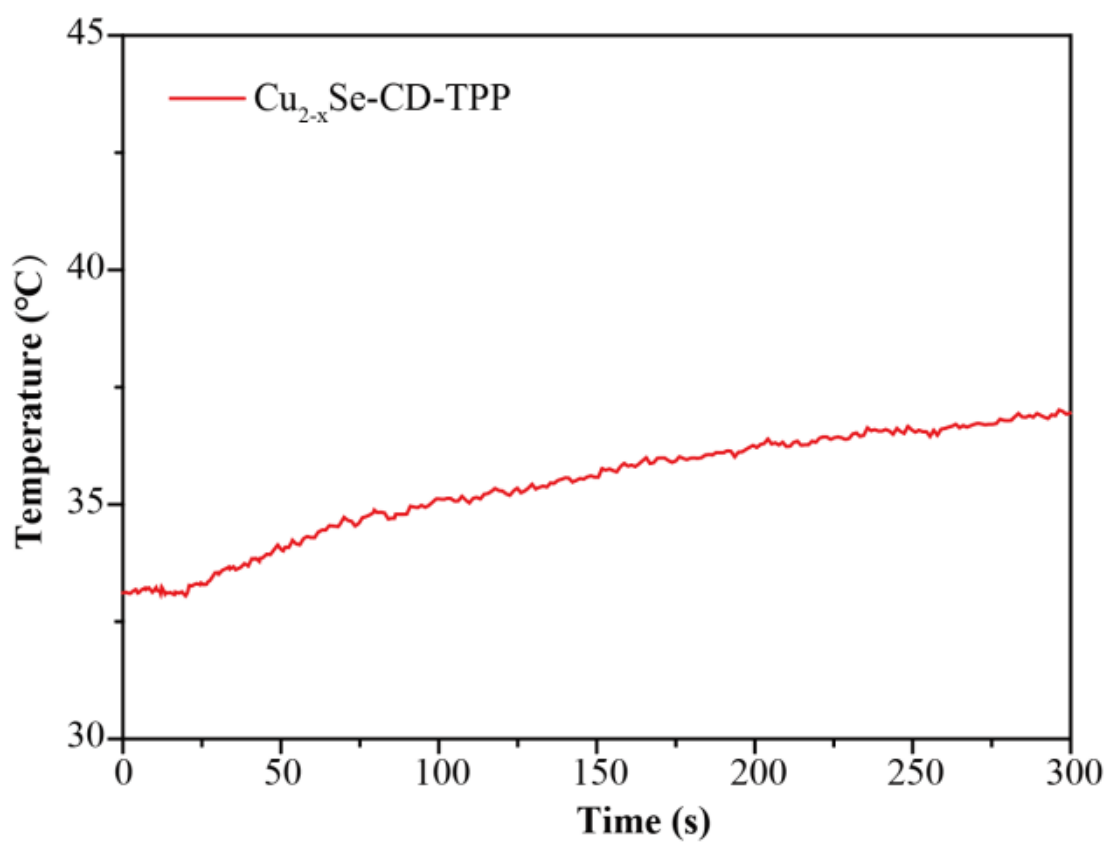

Figure S14. The corresponding real-time temperature profile of tumour-bearing mice (Cu_{2-x}Se-CD-TPP) under 650 nm laser irradiation (0.5 W cm^{-2}).
